# Chasing up and locking down the virus: Optimal pandemic interventions within a network

**DOI:** 10.1111/jpet.12604

**Published:** 2022-06-29

**Authors:** Michael Freiberger, Dieter Grass, Michael Kuhn, Andrea Seidl, Stefan Wrzaczek

**Affiliations:** ^1^ Economic Frontiers Program International Institute for Applied Systems Analysis (IIASA) Laxenburg Austria; ^2^ Vienna Institute of Demography Wittgenstein Centre (IIASA, ÖAW, Universität Wien) Vienna Austria; ^3^ Department of Business Decision and Analytics University of Vienna Vienna Austria

## Abstract

During the COVID‐19 pandemic countries invested significant amounts of resources into its containment. In early stages of the pandemic most of the (nonpharmaceutical) interventions can be classified into two groups: (i) testing and identification of infected individuals, (ii) social distancing measures to reduce the transmission probabilities. Furthermore, both groups of measures may, in principle, be targeted at certain subgroups of a networked population. To study such a problem, we propose an extension of the SIR model with additional compartments for quarantine and different courses of the disease across several network nodes. We develop the structure of the optimal allocation and study a numerical example of three symmetric regions that are subject to an asymmetric progression of the disease (starting from an initial hotspot). Key findings include that (i) for our calibrations policies are chosen in a “flattening‐the‐curve,” avoiding hospital congestion; (ii) policies shift from containing spillovers from the hotspot initially to establishing a symmetric pattern of the disease; and (iii) testing that can be effectively targeted allows to reduce substantially the duration of the disease, hospital congestion and the total cost, both in terms of lives lost and economic costs.

## 1 INTRODUCTION

Since the beginning of 2020 the virus SARS‐CoV‐2 has been spreading around the world at an incredible pace; and by May 2022 COVID‐19 (the disease induced by the virus) has cost more than 6.28 million lives.^1^ Before an effective and safe vaccination became available in sufficient quantities, which in case of COVID‐19 took about a year, mainly two instruments were available to governments: social distancing and testing.^2^

Social distancing is aimed at reducing the spread of the virus, and for the purpose of our paper, comprises—in a broad sense—measures such as locking down nonessential parts of the economy, travel restrictions, wearing masks in public, requiring distance between people in public spaces, and so on. Many of these measures have been used for decades to fight against infectious diseases (e.g., the plague, leprosy, or the Spanish flu) and can be implemented quickly but at potentially high costs to the economy and the individual (including disutility).

Testing and contract tracing, on the other hand, is essential to identify and isolate infected people and break infection chains. It is not surprising that many countries invested significantly into the development and deployment of such measures. While these relatively modern instruments are very useful for identifying and quarantining infected individuals and, thereby, allowing to gain control over the epidemic without locking down large parts of the population, tests and effective means of contact tracing have been in short supply, especially during the early phase of the pandemic and under circumstances of overwhelming infection rates.

The implementation of these measures varies substantially across countries. While social distancing and lockdown measures are commonly used with different intensity and focus and duration, testing tracing strategies are used very differently. South Korea, for example, concentrates on testing with a very efficient tracing strategy to detect infected people before they develop symptoms and are spreading around (*testing with efficient tracing*). Other countries, like Austria, try to test large parts of the population regularly and are implementing tests as a precondition for public entities like restaurants, events, cinemas, and so on (*unfocused testing*). As a third strategy a couple of countries are testing only people that develop symptoms. Regular testing of people without symptoms is not foreseen (*no testing*).

While there is a growing literature that evaluates social distance and lockdown measures (see the literature review later in this section), the question how testing and tracing should be implemented optimally and how it interacts with distance and lockdown measures has not been addressed so far. Another aspect that has attracted only scarce attention is the question whether these instruments should be applied uniformly across the population or should be varied according to the specific circumstances (in terms of localized infection rates and specific characteristics) of subgroups. This reflects the controversial public and political debate on whether or not targeted travel restrictions are appropriate, and whether social distancing should be focused in a way that shields only vulnerable groups but allows free interaction among others. Consequently our analysis then also links into a parallel debate on whether testing capacities should be focused on particular regional hotspots or certain subgroups of the population (accompanied by sophisticated tracing strategies), at least as long as test‐kits are in limited supply.

Ultimately these debates are about how to deal with heterogeneity across the population in two dimensions: (i) The state of disease progression, which typically varies across regions—and sometimes across population subgroups. (ii) The characteristics of subgroups of the population, where some are more vulnerable in a medical sense, that is, more prone to get infected and/or suffer from a severe course of the disease; and some may be more vulnerable economically, as captured by them facing higher costs of social distancing. In the following we discuss the general properties of these two dimensions of heterogeneity.

*Different regions*: As a pandemic, COVID‐19 has turned into a problem at global scale. This notwithstanding, countries and even regions differ in the starting date of the epidemic as well as in the pattern of epidemic progression. The advent of several waves of COVID‐19 for many countries also speaks evidence to the fact of cyclical patterns, where countries that had acquired control over the disease, lost it again due to spill‐overs from other countries. Another crucial issue concerning regionality are differences in the availability of intensive care capacities, with large variation even for countries that are close to each other.^3^ Similarly, intensive care units (ICU) may be regionally centralized, especially in small countries. For all of these reasons, policy measures may need to be regionally differentiated in their timing, as well as in their intensity.

*Heterogeneous population*: Scientists agree that COVID‐19 is a highly contagious disease that can take widely different courses. While it is mostly harmless for young and healthy people (although a number of long‐term effects are already recorded^4^), it can get very critical and even lead to death for old people or people with pre‐existing illnesses. Moreover, the social behavior of people of different age‐groups, different professions (people working in the health care sector, or as blue or white collar workers) is heterogeneous (social and working contacts are very different for children, blue and White collared workers, retired people). Hence, the population has to be subdivided in (disjoint) heterogeneous groups with respect to their infection risk, their probability to infect others and their susceptibility to experience a heavy course of the disease.

These arguments underline the importance of extending the existing epidemiological models in two dimensions: (a) to allow for the interaction of heterogeneous subgroups and for the targeting of policy‐instruments to certain groups; and (b) to allow for a more thorough analysis of the role of testing within a setting of optimal policy‐making. While attempts have been made in both directions, we will demonstrate in our literature review that these are to some extent patchy and focusing on specific contexts (e.g., analysis of exogenous variations in [nonoptimal] policies within decentralized economies). Thus, we believe to make headway with a coherent analysis of optimal policy‐making from a social planner perspective.

Additional compartments for quarantine and different courses of the disease (light, heavy, and detected). We consider the population to be distributed across a network (representing different regions or social groups, respectively) and model the diffusion of the virus between its nodes. We allow for heterogeneity across the network: Regions can be heterogeneous with respect to size and population density (implying different patterns of population interaction); social groups may differ with respect to parameters such as mortality and the number of interactions with other groups. Policy measures (testing and social distance/contact reduction) are allowed to be targeted in a way that minimizes the social cost of the pandemic. Tests are provided subject to a capacity constraint, and we consider variation in the effectiveness of testing (depending e.g., on whether or not tests can be targeted in the presence of contact tracing), which allows us to study the interaction of test efficacy with the level of contact reduction.

After providing and interpreting the first‐order conditions that govern optimal policy‐making and deriving properties of the optimal solution, we turn to numerical analysis. Here, we study the optimal control of a COVID‐19‐like disease^5^ spreading across three different regions. The regions are assumed to be symmetric and to differ only in the starting conditions (i.e., the disease spreads from an initial hotspot) such that the effects from the network structure are not dominated by regional asymmetries. We compare the optimal allocation and outcomes across four regimes: a regime of uncontrolled disease progression, a regime with lockdowns without any testing, and for two regimes with lockdown and testing, allowing for targeted testing in one case. While the social cost (in terms of economic loss, medical treatment costs, and value of lives lost) is reduced significantly by a relatively rigorous lockdown, the introduction of testing only yields significant cost reductions if tests can be targeted. Moreover, targeted testing changes the testing and lockdown strategy substantially. Whereas tests are allocated in a cyclical sequence across different regions/subgroups of the population, following high infection rates; targeted tests follow this pattern only initially but are subsequently shared equally across the subgroups. Policies are aimed at preventing spillover of the disease from the hotspot, but are then geared to the harmonization of the disease course across groups. For our parametrization, they are of a “flattening‐of‐the‐curve” type.

The remainder of the paper is organized as follows. Before introducing our model in greater detail we provide a brief overview of the recent literature on epidemiological models concerning the COVID‐19 pandemic in Section 2. Section 3 presents an extended SIR‐type model over a network. Section 4 continues with optimality conditions and properties of the optimal solution. A numerical scenario in which the disease spreads across three symmetric regions but starting from an asymmetric distribution of initial infections, is developed and analyzed in Section 5. Section 6 concludes. In the Supporting Information material, we present a second numerical scenario, involving a network composed of three population subgroups (blue‐collar workers facing high costs of lockdown, White‐collar workers facing low costs of lockdown, vulnerable individuals, for example, the elderly, with poorer health outlook upon infection).

## 2 LITERATURE REVIEW

Within this literature review, we restrict ourselves to a selection of papers that consider optimal policy making in a dynamic framework (i.e., in particular compartment models^6^ considered with optimal control theory) involving some heterogeneity across the population.

When dealing with the outbreak of an infectious disease, it is important to understand the impact of different policy measures. Optimal control theory provides tools to determine the optimal application of control instruments over the course of an epidemic to keep the direct and indirect harms of the disease as low as possible. While several papers consider only health related objectives (see e.g., Hansen & Day, 2011 or Bliman et al., 2021), there is also a growing literature that takes economic consequences of policy measures into account too. The optimal timing of a lockdown is studied in Caulkins et al. (2020), who found that the optimal duration of a lockdown decreases in its starting time. They analyze the trade off between economic damage and lost lives and identify conditions under which no lockdown, an intermediate and a permanent lockdown is optimal. Caulkins et al. (2021) extend this study by not only allowing the length of the lockdown to be optimally determined but also its intensity. Furthermore, they incorporate the impact of lockdown fatigue, that is, a declining adherence to policy measures related to their duration, on the optimal strategy. They find that the optimal solution is history‐dependent and that under certain conditions multiple lockdowns can be optimal. Caulkins et al. (2022) adjust the models of Caulkins et al. (2020, 2021) for more contagious virus mutations. Alvarez et al. (2021) analyze how the effectiveness of a lockdown, the fatality rate and the value of a statistical life affect the optimal lockdown policy. Not surpisingly, a low fatality rate or a low valued statistical life lead to a shorter, less intense lockdown, whereas a more effective lockdown increases the duration of the lockdown instead of decreasing it. The optimal lockdown policy is also studied in Federico and Ferrari (2021). They use an SIR framework where they assume the transmission rate to evolve according to a stochastic differential equation. They characterize three distinct phases in a pandemic: in the first phase, there should be no lockdown, while in the second phase it should be vigorous. In the third phase of the pandemic the lockdown should be moderate. The optimal containment measures to reduce the spreading of the disease, taking economic damage into account, have also been investigated by Aspri et al. (2020). Among other things, they emphasize the importance of testing which they find to enable the almost entire elimination of mortality at low economic costs in a one‐region/group model. El Ouardighi et al. (2021) take account of social fatigue and popular discontent when determining the optimal application of nonpharmaceutical interventions (mobility restrictions, isolation, and securing social interactions) over time.

Acemoglu et al. (2021) emphasize the importance of distinguishing between different risk groups when designing policy measures. Their results suggests a strict and long lockdown for the most vulnerable population group and a less strict lockdown for all other groups. Richard et al. (2021) focus on nonpharmaceutical interventions in an age‐structured optimal control framework. To minimize deaths and control costs, they recommend a strategy where control measures vary throughout the pandemic and are more intense for the older population. Bonnans and Gianatti (2020) consider an age‐structured population and analyze the optimal confinement strategy. The optimal vaccination strategy for different population groups is considered by Grundel et al. (2021). In their optimal control problem they minimize the amount of social distancing subject to the constraints that the number of people requiring ICU care remains beyond a certain threshold and that the availability of vaccines is limited. In principle, they recommend vaccinating people with high contact rates first, however, they also find situations in which vaccinating high‐risk groups is preferable. Angelov et al. (2021) use a distributed optimal control epidemiological model to study the impact of prioritization of age groups with respect to vaccination. They find that a random distribution of vaccines is efficient in reducing the overall number of infections, however, a lower mortality is obtained through prioritization of the elderly and of those groups with the highest contact rates. Fabbri et al. (2021) also take account of age‐structure within a SIR framework in a more theoretically oriented paper that provides sufficient optimality conditions.

To analyze whether or not policies should vary between different regions in Italy, Carli et al. (2020) extend their SIR‐based framework to a multiregion model. They focus on nonpharmaceutical interventions such as lockdowns and mobility restrictions between different regions. They propose a model predictive control approach to handle potential deviations of the predictive model from the actual development of a pandemic. They compare the impact of interventions that are uniformly applied to different Italian regions and interventions that are applied in a differentiated manner. The considered objective is to minimize economic and health‐related costs. They find that the model predictive control approach leads to substantially lower total costs than different benchmark control strategies and propose it as a suitable tool to mitigate future COVID‐19 waves. To determine the optimal vaccine distribution among different priority groups, Gamchi et al. (2021) embed a multiregion SIR model with multiple priority groups into a vehicle routing problem. By means of the weighted augmented epsilon constraint method, they study the trade‐off between the social costs and the costs of vaccinations and the fixed costs of vehicles. The main focus of their analysis is on the solution approach that combines the combinatorial VRP with an optimal control problem in a multiobjective framework. Numerical illustrations, however, confirm the importance of paying attention to high‐risk groups.

Different risk‐groups, testing, and the impact of social distancing measures are also considered in Gollier (2020). In this paper, confinement strategies are compared where the degree of confinement is either constant over time or can assume two different levels, depending on the number of infected. Our paper extends this study by optimally determining the degree of testing and contact‐reducing measures.

Gori et al. (2021) focus on the economic impact of the COVID‐19 pandemic and analyse the impact of testing to avoid costly lockdowns in a framework that does not differentiate between different regions or population groups, but they explicitly consider the dynamics of capital. The duration of a lockdown is determined by a feedback rule, which depends, in particular, on the prevalence. Lockdown and testing intensity are determined optimally, however, they are assumed to be constant over time. In our paper, we allow the intensity of social distancing measures and testing to vary over time.

## 3 SIR‐TYPE MODEL

For the epidemic dynamics, we extend the well‐known SIR model (see Kermack & McKendrick, 1927) over a network Ω. The network consists of ∣Ω∣ (denoting the cardinality of Ω) network nodes representing different population groups or distribution according to space (i.e., geographical location).^7^ At every node j∈Ω we consider the following seven compartments: susceptible individuals $S_{j}\left(t\right)$, infected individuals without or with light symptoms $L_{j}\left(t\right)$, infected individuals without or with light symptoms that have been detected by a positive test $D_{j}\left(t\right)$, infected individuals with heavy symptoms $H_{j}\left(t\right)$, recovered individuals having never been detected $R_{j}^{L}\left(t\right)$, recovered individuals having been detected $R_{j}^{D}\left(t\right)$, and deceased individuals $M_{j}\left(t\right)$.^8^ Table 1 summarizes the seven compartments of our network model and illustrates the refinements in comparison to the classical SIR model.

**Table 1:** Compartments (state variables) of the extended SIR model

Symbol	Description	Abbreviation	Classical SIR model
$S_{j}\left(t\right)$	Susceptibles		S(t) susceptibles
$L_{j}\left(t\right)$	Infected without or with light symptoms	Light cases	
$D_{j}\left(t\right)$	Diagnosed light cases detected through testing	Diagnosed cases	I(t) infected
$H_{j}\left(t\right)$	Infected with heavy symptoms (hospitalized)	Heavy cases	
$R_{j}^{L}\left(t\right)$	Recovered light cases not detected during infection	Recovered light cases	R(t) recovered
$R_{j}^{D}\left(t\right)$	Recovered heavy and diagnosed light cases	Recovered diagnosed cases	
$M_{j}\left(t\right)$	Deceased		Outflow

*Note*: The first three columns show the mathematical symbol, the description and the abbreviation used in the paper. The last column relates the compartments to those of the classical SIR model.

**Figure 1:**
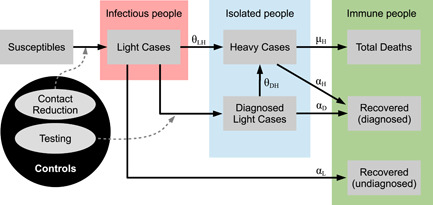
Flow chart of the SIR‐type model. Gray boxes: compartments, gray ellipses: controls, black arrows: flows between the compartments, dashed arrows: flows that can be changed by controls.

Figure 1 illustrates the flows between the different compartments on each node j∈Ω of the network. The compartments in the gray boxes are grouped into the susceptible population (as a source of new infections), the infected population (red—responsible for new infections), the quarantined population (blue—infected population without contact to the susceptible population), and the immune population (green—unable to infect susceptibles and to acquire an infection, i.e., the sink). The flows generally follow the different courses of progression of the infection. Susceptibles can get infected and initially only suffer light symptoms (in every scenario), that is, become a light case. Subsequently, there are three potential paths of progression: (i) a light case can recover at rate $\alpha_{L}$, (ii) a light case can develop more severe symptoms and escalate to a heavy case in need of hospital treatment, (iii) a light case can be detected and diagnosed by being tested. In case (i) the individual becomes part of the undiagnosed recovered compartment, which are subsequently assumed to be immunized.^9^ A heavy case in (ii) is automatically isolated in the hospital and does not contribute to the spread of the infection any more. A heavy case recovers at rate $\alpha_{H}$ and dies at rate $\mu_{H}$. Accordingly, the deceased are counted in the compartment of total deaths, while upon recovery individuals are counted as diagnosed recovered cases. The diagnosed light cases in (iii) also do not contribute to the infection dynamics as they are isolated (e.g., in home isolation). Being isolated they can either recover or escalate to a heavy case analogously to the undetected light cases. However, their recovery and escalation rates may differ, as an early diagnosis allows for potential treatment and the prevention of escalation for these specific cases.

The controls are denoted in the gray ellipses and consist of contact reduction measures and testing efforts. While contact reduction impacts the flow from susceptibles to light cases, testing efforts affect the flow from light cases to diagnosed light cases. Both of these channels are indicated with dashed arrows in Figure 1.

The network structure of our framework allows us to model a population that is heterogeneous with respect to its spatial distribution and its epidemiologically relevant characteristics, such as profession, infection and mortality risk, system relevance (among others). Furthermore, these subgroups interact according to their “interconnections” along the arcs^10^ (i.e., individuals move between the compartments of the same node,^11^ where the size of the flow possibly depends on the interaction with other nodes), which enables us to describe complex infection dynamics across different subpopulations. For simplicity (and due to the relatively short time horizon of the pandemic) we abstract from modeling population inflow (i.e., births or migration) and natural outflow (i.e., non‐COVID‐19 mortality). Following from the network structure of Ω and the state variables presented in Figure 1 at each node, our framework consists of 7×∣Ω∣ state variables in total.

In the following sections, we will present the dynamics of each state variable, the policy maker's objective and cost functions, and we conclude by presenting the complete problem formulation.

### 3.1 Dynamical system

For the presentation of the state dynamics we follow the flows between the states as in Figure 1.

*Susceptibles* at node j∈Ω are assumed to be in contact with other susceptibles, light cases or recovered individuals (diagnosed cases are isolated at home and heavy cases are isolated in a hospital) of any node k∈Ω (i.e., along an undirected arc connecting nodes j and k). Thus susceptibles can only be infected by a contact with an undetected light case (from any node). The probability that a random contact with an individual from node k is infectious corresponds to the share of light cases in the nonisolated population, that is, $\frac{L_{k}}{S_{k}+L_{k}+R_{k}^{L}+R_{k}^{D}}$. Note that $D_{k}$ and $H_{k}$ appear neither in the nominator nor in the denominator of this probability, since diagnosed and heavy cases are isolated. The combination of the contact rate of susceptibles in node j with individuals of node k and the rate at which susceptibles of node j get infected by light cases of node k (in case of contact) at time t is denoted by $u_{k,j}\left(t\right)$. From now on we will refer to it as transmission rate from k to j. Consequently, the dynamics for the number of susceptibles at node j can be described by

(1) $${\dot{S}}_{j}=-S_{j}\cdot \sum_{k\in \Omega }\left(u_{k,j}\cdot \frac{L_{k}}{S_{k}+L_{k}+R_{k}^{L}+R_{k}^{D}}\right),\;S_{j}(0)=S_{j0}.$$

Within our framework we propose that the (nonnegative) transmission rate $u_{k,j}\left(t\right)$ is a control variable of the decision‐maker, which is bounded from above by the natural transmission rate $\beta_{k,j}$. Specifically $u_{k,j}\left(t\right)$ can be directly chosen as a “target transmission rate” in the dynamics subject to nonlinear control costs in the objective function (as discussed in Section 3.2).^12^ While this is equivalent to the modeling of the decision‐maker choosing an effort (at diminishing returns) to control transmission, the direct choice of a transmission rate is more directly aligned with policy‐makers setting targets in terms of reducing the effective reproduction number (which depends on the transmission rate) below a certain threshold.^13^

The number of *light cases* in node j∈Ω increases with the inflow of newly infected susceptibles (outflow of (1)) and decreases at the (exogenous) rate $\theta_{LH}\left(j\right)$ at which light cases turn into heavy cases, at the (exogenous) rate $\alpha_{L}\left(j\right)$ at which light cases recover without treatment, and at the rate at which light cases are detected and quarantined. The detection rate $\frac{v_{j}}{L_{j}+\kappa \left(S_{j}+R_{j}^{L}\right)}$ sets the number of performed tests $v_{j}\left(t\right)$ (from now on referred to as testing effort at node j∈Ω) in relation to the number of individuals which are potentially infected and still undetected $L_{j}+\kappa \left(S_{j}+R_{j}^{L}\right)$. Although the social planer knows/estimates the number of light cases, he cannot identify who is infected without testing. The parameter κ∈[0,1], which is assumed to be an exogenous parameter, corresponds to different testing capabilities/efficacies. For κ close to zero, the decision‐maker is able to almost perfectly identify the light cases a priori, that is, already before testing. This scenario corresponds to the assumption of all infected showing specific symptoms such that testing can be restricted to these individuals. For κ close to one, the decision‐maker lacks such information and most infections develop asymptomatically. Without any form of contact tracing, the planer can only test randomly across the relevant population consisting of light cases $L_{j}$, of susceptibles $S_{j}$, and of recovered individuals $R_{j}^{L}$, who were not diagnosed in the past.^14^ However, even in case of high rates of asymptomatic infections, contact tracing facilitates the identification of potential infections and enables better targeting of tests.^15^ In our framework this corresponds to interior values of κ∈(0,1), reflecting scenarios of imperfect testing. The dynamics for the number of light cases then read

(2) $${\dot{L}}_{j}=S_{j}\cdot \sum_{k\in \Omega }\left(u_{k,j}\cdot \frac{L_{k}}{S_{k}+L_{k}+R_{k}^{L}+R_{k}^{D}}\right)-\theta_{LH}(j)L_{j}-\alpha_{L}(j)L_{j}\;-\frac{v_{j}}{L_{j}+\kappa \left(S_{j}+R_{j}^{L}\right)}L_{j},\;L_{j}(0)=L_{j0}.$$

*Diagnosed light cases* at node j∈Ω are placed in isolation and do not further contribute to the spread of the disease. These individuals can either recover at (exogenous) rate $\alpha_{D}\left(j\right)$ or turn into heavy cases at an (exogenous) rate $\theta_{DH}\left(j\right)$. We allow these rates to (possibly) differ from the corresponding transition rates for undetected light cases, reflecting the effects of possible treatment and better monitoring of diagnosed cases. The number of diagnosed cases increases at the rate at which tests are identifying light cases (as explained above). Putting things together, the dynamics of the diagnosed cases at node j∈Ω read

(3) $${\dot{D}}_{j}=\frac{v_{j}}{L_{j}+\kappa \left(S_{j}+R_{j}^{L}\right)}L_{j}-\alpha_{D}(j)D_{j}-\theta_{DH}(j)D_{j}\;,\;D_{j}(0)=D_{j0}.$$

*Heavy cases* are assumed to be diagnosed and treated in a hospital. Therefore, they do not contribute to the spread of the virus any more. Individuals suffering a heavy course enter through the escalation of light infections (detected or undetected) and leave the hospital either through death at rate $\mu_{H}\left(\bar{H_{j}},j\right)$ or recovering at rate $\alpha_{H}\left(\bar{H_{j}},j\right)$. As we will explain further on below, both rates may depend on hospital congestion, as measured by $\bar{H_{j}}$ (see Equation 5) at node j∈Ω. The dynamics for heavy cases are then given by

(4) $${\dot{H}}_{j}=\theta_{LH}(j)L_{j}+\theta_{DH}(j)D_{j}-\alpha_{H}(\bar{H_{j}},j)H_{j}-\mu_{H}(\bar{H_{j}},j)H_{j},\;H_{j}(0)=H_{j0}.$$

To account for potential congestion effects in the medical sector at node j∈Ω the recovery rate $\alpha_{H}\left(\bar{H_{j}},j\right)$ and the mortality rate $\mu_{H}\left(\bar{H_{j}},j\right)$ are assumed to depend on the aggregated variable $\bar{H_{j}}$, as defined by

(5) $$\bar{H_{j}}=\sum_{k\in \Omega }H_{k}f_{H}\left(j,k\right).$$

Here, the function $f_{H}\left(j,k\right)$ relates heavy cases from node k to hospital (or, indeed, intensive care) utilization at node j. If, for instance, the network describes social strata of a population all of which are treated within the same hospital, we have $f_{H}\left(j,k\right)=1$ for j,k∈Ω and thus $\bar{H_{j}}=\bar{H}=\sum_{k\in \Omega }H_{k}$ for j∈Ω. If, in contrast, the network describes different regions with segregated hospitals, then $f_{H}\left(j,k\right)=1$ for k=j and zero otherwise, implying that $\bar{H_{j}}=H_{j}$. Of course, other forms of $f_{H}\left(j,k\right)$ can be assumed, reflecting situations in which (some) patients from one region may be treated in another region.

*Recovered light and diagnosed cases*, as well as the *deceased* ($R_{j}^{L},R_{j}^{D}$, and $M_{j}$ resp.) at node j collect the outflows from the corresponding compartments. Thus, the dynamics read

(6) $${\dot{R}}_{j}^{L}=\alpha_{L}\left(j\right)L_{j},\;R_{j}^{L}\left(0\right)=R_{j0}^{L},$$

(7) $${\dot{R}}_{j}^{D}=\alpha_{D}\left(j\right)D_{j}+\alpha_{H}\left(\bar{H_{j}},j\right)H_{j},\;R_{j}^{D}\left(0\right)=R_{j0}^{D},$$

(8) $${\dot{M}}_{j}=\mu_{H}\left(\bar{H_{j}},j\right)H_{j},\;M_{j}\left(0\right)=M_{j0}.$$

### 3.2 Costs and objective function

The decision‐maker has two control variables: the transmission rate $u_{k,j}\left(t\right)$ between light cases at node k∈Ω and susceptibles at node j∈Ω (see Equation 1) and the testing effort $v_{j}\left(t\right)$ at node j∈Ω (see Equation 2). Both controls aim at containing the epidemic and lowering the number of deaths. Testing and tracing is a scarce resource. In spring 2020, COVID‐19 testing kits and laboratory capacities were scarcely available and had to be rationed. To account for limited availability of testing, we include a constraint on the corresponding control, that is,

(9) $$\sum_{j\in \Omega }v_{j}\left(t\right)\leq \bar{V}\left(t\right),$$

where $\bar{V}\left(t\right)$ is the maximum number of available tests for the entire network. The dependency on time t reflects that supply may grow over time.^16^

Let $C_{U}\left(u_{k,j}\left(t\right),t,k,j\right)$ denote the cost of curtailing the transmission rate $u_{k,j}\left(t\right)$ from node k to j (at t) to a level below its unrestricted (natural) level $\beta_{k,j}$. Thus, we assume $C_{U}\left(u_{k,j}\left(t\right),t,k,j\right)$ to be convex decreasing (i.e., a higher cost for a lower transmission rate) for all $0\leq u_{k,j}\left(t\right)\leq \beta_{k,j}$. Without any intervention, that is, for $u_{k,j}\left(t\right)=\beta_{k,j}$ the cost is zero. The cost for testing effort at node j∈Ω is denoted by $C_{V}\left(v_{j}\left(t\right),t,j\right)$ and is convex increasing. Again, we assume that $v_{j}=0$ generates no cost. We can then summarize the properties of the cost functions related to the control variables (j,k∈Ω) as follows:

(10a) $$\frac{\partial C_{U}\left(\cdot \right)}{\partial u_{k,j}}<0,\;\frac{\partial^{2}C_{U}\left(\cdot \right)}{\partial u_{k,j}^{2}}\geq 0,\;C_{U}\left(\beta_{k,j},t,k,j\right)=0,$$

(10b) $$\frac{\partial C_{V}\left(\cdot \right)}{\partial v_{j}}>0,\;\frac{\partial^{2}C_{V}\left(\cdot \right)}{\partial v_{j}^{2}}\geq 0,\;C_{V}\left(0,t,j\right)=0.$$

The occurrence of heavy cases (subsumed in the $H_{j}$ compartment, j∈Ω) leads to two types of (monetary) costs. First, (by definition) all heavy cases have to be treated in a hospital, some of them even in an ICU, implying medical treatment costs $C_{M}\left(H_{j}\left(t\right),t,j\right)$ associated with type j patients at time t. Again we assume these costs to be convex increasing in $H_{j}\left(t\right)$. Second, some of the heavy cases do not survive. The lost lives from group j are weighted by the statistical value of life Ψ, which we assume to be equal across all nodes.

The policy‐maker aims at minimizing total cost, composed of the costs for controlling the transmission rates, the costs for testing, the treatment costs and the value of lost lives, over the course of the pandemic from its onset at t=0 to its end at t=T which for the purpose of fixing ideas we identify with the point in time at which an effective and safe vaccine is universally available.^17^ Thus the objective function can be formulated as

(11) $$\min_{\begin{array}{c} v_{j}\left(t\right)\geq 0\\ 0\leq u_{k,j}\left(t\right)\leq \beta_{k,j} \end{array}}\int_{0}^{T}e^{-\rho t}\left[\sum_{j\in \Omega }(C_{M}\left(H_{j}\left(t\right),t,j\right)+\mu_{H}\left(\bar{H_{j}}\left(t\right),j\right)H_{j}\left(t\right)\cdot \Psi \;+\;\left.C_{V}\left(v_{j}\left(t\right),t,j\right))\;+\sum_{\left(k,j\right)\in \Omega^{2}}C_{U}\left(u_{k,j}\left(t\right),t,k,j\right)\right]dt\;+\;\right.e^{-\rho T}S\left(X\left(T\right),T\right),$$

where ρ≥0 is the discount rate and where the salvage value function S(X(T),T) contains all follow‐up costs of the pandemic. Here X(T) is used as an abbreviation and denotes a vector of all seven compartments across the network. For the salvage value we assume the same costs as for the planning horizon, but since the pandemic is over after T, there is no need for further controls (implying zero control costs). Moreover, our assumption of unlimited availability of an effective and safe vaccine implies that susceptibles cannot get infected any more.^18^^,^ 19 Hence, the relevant compartments can be reduced to the light, the diagnosed and the heavy cases, as only these individuals cause costs until there are no cases left. The salvage value function can then be written as follows:

(12) $$S\left(X\left(T\right),T\right)≔\int_{T}^{\infty }e^{-\rho \left(t-T\right)}\left[\sum_{j\in \Omega }C_{M}\left(H_{j}\left(t\right),t,j\right)+\mu_{H}\left(\bar{H_{j}}\left(t\right),j\right)H_{j}\left(t\right)\cdot \Psi \right]dt,$$

where the states evolve (without further control) according to (j∈Ω)

(13a) $${\dot{L}}_{j}=-\left(\theta_{LH}\left(j\right)+\alpha_{L}\left(j\right)\right)L_{j},$$

(13b) $${\dot{D}}_{j}=-\left(\theta_{DH}\left(j\right)+\alpha_{D}\left(j\right)\right)D_{j},$$

(13c) $${\dot{H}}_{j}=\theta_{LH}\left(j\right)L_{j}+\theta_{DH}\left(j\right)D_{j}-\left(\alpha_{H}\left(\bar{H_{j}},j\right)+\mu_{H}\left(\bar{H_{j}},j\right)\right)H_{j},$$

(13d) $$\bar{H_{j}}=\sum_{k\in \Omega }H_{k}f_{H}\left(j,k\right).$$

### 3.3 Complete problem

Putting things together the policy‐maker faces the following finite time optimal control problem:

(14a) $$\min_{\begin{array}{c} v_{j}(t)\geq 0\\ 0\leq u_{k,j}(t)\leq \beta_{k,j} \end{array}}\int_{0}^{T}e^{-\rho t}\left[\sum_{j\in \Omega }(C_{M}(H_{j},t,j)+\mu_{H}(\bar{H_{j}},j)H_{j}(t)\cdot \Psi +C_{V}(v_{j},t,j))\;+\sum_{(k,j)\in \Omega^{2}}C_{U}(u_{k,j},t,k,j)\right]dt+e^{-\rho T}S(X(T),T)$$

(14b) $${\dot{S}}_{j}=-S_{j}\cdot \sum_{k\in \Omega }\left(u_{k,j}\cdot \frac{L_{k}}{S_{k}+L_{k}+R_{k}^{L}+R_{k}^{D}}\right)$$

(14c) $${\dot{L}}_{j}=S_{j}\cdot \sum_{k\in \Omega }\left(u_{k,j}\cdot \frac{L_{k}}{S_{k}+L_{k}+R_{k}^{L}+R_{k}^{D}}\right)-\theta_{LH}(j)L_{j}-\alpha_{L}(j)L_{j}-\frac{v_{j}}{L_{j}+\kappa \left(S_{j}+R_{j}^{L}\right)}L_{j}$$

(14d) $${\dot{D}}_{j}=\frac{v_{j}}{L_{j}+\kappa \left(S_{j}+R_{j}^{L}\right)}L_{j}-\alpha_{D}\left(j\right)D_{j}-\theta_{DH}\left(j\right)D_{j}$$

(14e) $${\dot{H}}_{j}=\theta_{LH}\left(j\right)L_{j}+\theta_{DH}\left(j\right)D_{j}-\alpha_{H}\left(\bar{H_{j}},j\right)H_{j}-\mu_{H}\left(\bar{H_{j}},j\right)H_{j}$$

(14f) $${\dot{R}}_{j}^{L}=\alpha_{L}\left(j\right)L_{j}$$

(14g) $${\dot{R}}_{j}^{D}=\alpha_{D}\left(j\right)D_{j}+\alpha_{H}\left(\bar{H_{j}},j\right)H_{j}$$

(14h) $${\dot{M}}_{j}=\mu_{H}\left(\bar{H_{j}},j\right)H_{j}$$

(14i) $$\sum_{j\in \Omega }v_{j}\left(t\right)\leq \bar{V}\left(t\right),\bar{H_{j}}=\sum_{k\in \Omega }H_{k}f_{H}\left(j,k\right),$$

where S(X(T),T) is defined by (12) and (13), and where $X\left(0\right)=X_{0}$.

## 4 PROPERTIES OF THE OPTIMAL SOLUTION

Within this section, we first formulate the first‐order conditions for the control variables (i.e., target transmission rates and number of performed tests) and provide an intuitive understanding (Section 4.1). Second, we are deriving general properties that help to understand the optimal solution (Section 4.2).

### 4.1 Optimal allocation

From the Maximum Principle we can formulate the Hamiltonian and derive first‐order optimality conditions and adjoint equations for the costate variables (see Grass et al., 2008). The whole set of derivations is relegated to the Supporting Information: Online Appendix. Within this section, we provide economic intuition by discussing the first‐order conditions and some theoretical results that enhance the understanding of the optimal solution. A thorough discussion of the shadow prices relating to the different state variables, which are frequently part of the FOCs, can be found in the Supporting Information: Online Appendix.

The first‐order conditions for the control variables (in the interior of their respective feasible region) can be formulated as follows (j,k∈Ω):

(15a) $$\frac{\partial C_{U}\left(u_{k,j},t,k,j\right)}{\partial u_{k,j}}=\left(\lambda_{L_{j}}-\lambda_{S_{j}}\right)S_{j}\cdot \frac{L_{k}}{S_{k}+L_{k}+R_{k}^{L}+R_{k}^{D}}$$

(15b) $$\frac{\partial C_{V}\left(v_{j},t,j\right)}{\partial v_{j}}=\left(\lambda_{D_{j}}-\lambda_{L_{j}}\right)\cdot \frac{L_{j}}{L_{j}+\kappa \left(S_{j}+R_{j}^{L}\right)}+\Lambda ,$$

where $\lambda_{x}$ denotes the shadow price of state variable x; and where Λ denotes the Lagrangian multiplier relating to the constraint on testing capacity (9). The first set of Equation (15a) determines the optimal target transmission rates (for infections from node k to node j), the second set (15b) refers to optimal testing efforts at node j.^20^

Equation (15a) equates the cost of a marginal reduction in the target transition rate with the benefit of lowering the probability of an additional infection at node j.^21^ The latter consists of the product of (i) the value of avoiding an infection at node j, as measured by the difference between $\lambda_{L_{j}}$ and $\lambda_{S_{j}}$; (ii) the number of susceptibles at node j; and (iii) the probability that the contact of a susceptible of node j at node k is infectious (i.e., $\frac{L_{k}}{S_{k}+L_{k}+R_{k}^{L}+R_{k}^{D}}$).

Analogously, Equation (15b) equates the cost of a marginal increase in testing effort with the marginal benefit of raising the probability of identifying and isolating a light case at node j. The latter consists of the product of (i) the value of identifying a light case at node j, as given by the difference between $\lambda_{D_{j}}$ and $\lambda_{L_{j}}$; and (ii) the probability that a light case is detected by the test (i.e., $\frac{L_{j}}{L_{j}+\kappa \left(S_{j}+R_{j}^{L}\right)}$). The Lagrangian multiplier Λ is zero as long as the testing capacity is not exhausted (see complementary slackness condition as formulated in optimality conditions in the Supporting Information: Online Appendix). In case the testing capacity is in full usage, the multiplier turns negative, implying that the marginal benefit exceeds the marginal cost of testing at all nodes. Under such a situation of rationing, tests are allocated such that the gap between marginal benefits and costs is equilibrated across nodes. This implies, in particular, that all else equal more tests tend to be allocated to those nodes that either exhibit a larger number of light cases $L_{j}$, as this raises the probability of detecting a light case and, thus, the efficacy of testing; or exhibit a higher value of detection, as is true, for example, for nodes at which infections are very harmful.

Sufficiency conditions are difficult to deal with in our model. The Mangasarian and Arrow sufficiency conditions (see Grass et al., 2008) are not promising for our model due to nonconvexities in the constraints; a feature which is well‐known for models with SIR dynamics (see Gersovitz & Hammer, 2004). Another possibility is presented in Goenka et al. (2021) (see also the discussion in Boucekkine et al., 2021), who prove local optimility by adopting the Leitmann–Stalford sufficiency conditions (see Leitmann & Stalford, 1971). However, the additional compartments, as well as the network structure of our model, imply considerable complications so the proposed method is not applicable.

For the numerical solution, we adopted a gradient‐based optimization method developed by Veliov, 2003 for age‐structured optimal control problems. Following gradual improvements along the direction of the (negative) gradient assures that the numerical solution does not correspond to a maximum of the objective function, which can be the case for techniques using the first‐order conditions directly. We are aware that this approach still poses the risk of termination of the algorithm in a local optimum. However, we checked our numerical solution for a variety of initial guesses of the control profiles to combat this problem.

### 4.2 Properties

The first‐order conditions (15a) and (15b) define the values of the control variables, target transmission rates, and testing effort, in the optimal solution in each node j. However, it is not directly clear how they are related. The following Proposition investigates how the optimal target transmission rates are connected to each other and highlights the network aspect of our framework.

Proposition 1 Consider the full finite‐time optimal control model (14) and assume that an optimal solution exists. If the transmission rate from any two nodes $k_{1}$ and $k_{2}$ to nodes $j_{1}$ and $j_{2}$, respectively does not lie on the boundary, the ratio of the marginal costs of controlling transmissions from region $k_{1}$ as opposed to region $k_{2}$ into any third region $j=j_{1},j_{2}$ depends only on the ratio of the shares of light cases among the nonisolated population in $k_{1}$ as opposed to $k_{2}$, that is,

(16) $$\frac{\frac{\partial C_{U}\left(u_{k_{1},j_{1}},t,k_{1},j_{1}\right)}{\partial u_{k_{1},j_{1}}}}{\frac{\partial C_{U}\left(u_{k_{2},j_{1}},t,k_{2},j_{1}\right)}{\partial u_{k_{2},j_{1}}}}=\frac{\frac{\partial C_{U}\left(u_{k_{1},j_{2}},t,k_{1},j_{2}\right)}{\partial u_{k_{1},j_{2}}}}{\frac{\partial C_{U}\left(u_{k_{2},j_{2}},t,k_{2},j_{2}\right)}{\partial u_{k_{2},j_{2}}}}=\frac{\frac{L_{k_{1}}}{S_{k_{1}}+L_{k_{1}}+R_{k_{1}}^{L}+R_{k_{1}}^{D}}}{\frac{L_{k_{2}}}{S_{k_{2}}+L_{k_{2}}+R_{k_{2}}^{L}+R_{k_{2}}^{D}}},\forall \;\;k_{1},k_{2},j_{1},j_{2}\in \Omega .$$

Since the transmission rates do not lie on the boundary the corresponding first‐order conditions for $u_{k_{1},j_{1}}\left(t\right),u_{k_{2},j_{1}}\left(t\right),u_{k_{1},j_{2}}\left(t\right)$, and $u_{k_{2},j_{2}}\left(t\right)$ are defined by (15a). Dividing one by the other (for fixed $j_{1},j_{2}$, respectively) proves the above assertion.  □

Therefore, the ratio of the marginal cost of the target transmission rates from any two regions of origin into any third region is equalized across all receiving regions. In other words, the marginal rate of transformation in respect to controlling the spread of infections from any two regions of origin is equalized across the network. Furthermore, the marginal rate of transformation does not depend on any of the shadow prices, but only on the share of light cases among the nonisolated population within the two regions of origin. Interestingly, this means that the optimal target transmission rate at every node (in relation to the other nodes) is set only upon the current state of the pandemic course without considering the dynamics. Consequently, it is enough to set only one target transmission rate optimally with respect to the dynamic effects included in the adjoint variables (according to 15a). All other target transmission rates that govern infections in the same node can be set without considering the dynamic effects.

In particular, the ratio of marginal costs of the optimal transmission rates from two different nodes $k_{1}$ and $k_{2}$ to a common neighboring node j does not depend on the epidemic situation at node j. On the other hand, a small transformation of Equation (16) directly leads to the following corollary.

Corollary 2 Under the assumptions of Proposition 1 it holds that

$$\frac{\frac{\partial C_{U}\left(u_{k_{1},j_{1}},t,k_{1},j_{1}\right)}{\partial u_{k_{1},j_{1}}}}{\frac{\partial C_{U}\left(u_{k_{1},j_{2}},t,k_{1},j_{2}\right)}{\partial u_{k_{1},j_{2}}}}=\frac{\frac{\partial C_{U}\left(u_{k_{2},j_{1}},t,k_{2},j_{1}\right)}{\partial u_{k_{2},j_{1}}}}{\frac{\partial C_{U}\left(u_{k_{2},j_{2}},t,k_{2},j_{2}\right)}{\partial u_{k_{2},j_{2}}}}\;\forall \;\;k_{1},k_{2},j_{1},j_{2}\in \Omega .$$

Corollary 2 shows that the ratio of marginal costs of the optimal transmission rates from any given node k to the same two neighboring nodes $j_{1}$ and $j_{2}$ is identical for all possible k. Thus the marginal rate of transformation in terms of protecting groups $j_{1}$ and $j_{2}$ is equalized across all nodes of infection origin k.

For an analytical relationship between the optimal testing strategy and the optimal target transmission rates, we will assume an interior solution for testing, where additionally the testing capacity constraint is not binding. We can derive the following equation for testing in node j and the optimal target transmission rate within node j by dividing Equations (15a) and (15b).

(17) $$\frac{\left(\frac{\partial C_{U}\left(u_{j,j},t,j,j\right)}{\partial u_{j,j}}\right)/S_{j}}{\frac{\partial C_{V}\left(v_{j},t,j\right)}{\partial v_{j}}}=\frac{\left(\lambda_{L_{j}}-\lambda_{S_{j}}\right)}{\left(\lambda_{D_{j}}-\lambda_{L_{j}}\right)}\cdot \frac{L_{j}+\kappa \left(S_{j}+R_{j}^{L}\right)}{L_{j}+S_{j}+R_{j}^{L}+R_{j}^{D}}.$$

On the left‐hand side we obtain the ratio of the marginal costs of a change in the transmission rate per susceptible, that is, the population which is directly affected by the transmission, and the marginal costs of additional tests. On the right side we first have the ratio of the (shadow) value of an infection relative to the (shadow) value of a detection via testing. This ratio is multiplied with the share of the potential testing pool in the active (nonisolated) population. Note that the number of infections $L_{j}$ does not appear as an isolated term in Equation (17), but only as part of the active population (denominator) and the testing pool (nominator).

The share of the testing pool in the active population plays a crucial rule for the relative allocation of resources toward lockdown measures or testing, but it also varies over time. For COVID‐19‐like diseases (see the numerical solution in Section 5) the variations of nominator and denominator are relatively small. The active population at each point in time is equal to the total population without the total number of fatalities $M_{j}$ and isolated cases ($D_{j}+H_{j}$). The latter two terms are orders of magnitude smaller than the total population and as a result the active population is not subject to large variations over time. A similar argument holds for the testing pool, but we have to account for the number of recovered diagnosed cases. In case of high numbers of detections of light cases (through effective or high‐volume testing) we would expect the size of the testing pool to decrease over time. A more substantial impact on the ratio $\frac{L_{j}+\kappa \left(S_{j}+R_{j}^{L}\right)}{L_{j}+S_{j}+R_{j}^{L}+R_{j}^{D}}$ results from the testing efficiency κ. For highly effective testing (i.e., κ≈0) this ratio would be close to zero (again the number of light cases is at least one order of magnitude smaller then the active population for COVID‐19‐like diseases) and consequently imply more tests and less lockdown measures (in case of convex costs for both measures). For ineffective testing (κ≈1), the ratio is close to one and conversely suggests a greater focus on lockdown measures as opposed to testing.

## 5 NUMERICAL ANALYSIS

In this section, we apply the model to study the disease dynamics and the impact of optimal policy‐making within a network composed of three nodes. Specifically, we consider three regions (i.e., a geographic network) that interact with one another and share resources with respect to tests and hospital capacities. The regions are homogeneous in their structure but differ in initial infection numbers. In the Supporting Information: Online Appendix we present an alternative application of our framework, where we assess the potential benefits of targeting social distancing, lockdown, and testing measures at specific subgroups of a population distinguished by their economic and demographic make‐up.

We assume that the pandemic is terminated by a vaccine that becomes available after 1 year (i.e., T=360 days) and that no transmission takes place afterward. We present the dynamic development until Day 400, as this fully covers the dynamics underlying the salvage value function in Equations (12) and (13). As we try to focus on the identification of network effects in our model, we assume the three regions to be identical regarding their characteristics and their interactions to be symmetric. Introducing specific heterogeneity in some aspects could obscure effects resulting purely from the network structure. Using this approach we can attribute all heterogeneous measures and developments to differences in the initial conditions of the three regions. Specifically, we impose:

(*G*1) The regions are identical in population size. With the total population being normalized to 1, we then have $N_{i}\left(t\right)=1∕3$ for all t and i∈Ω.
(*G*2) Epidemiological and cost parameters (i.e., regeneration, escalation, and mortality rates; costs for treatment, testing, implementing the target transmission rate, and lost lives) are identical across regions, see Table 2.
(*G*3) Hospital capacities are shared between the regions, which means that heavy cases can be admitted to hospitals in other regions if the local capacities are exhausted, that is, $f_{H}\left(j,k\right)\equiv 1$.
(*G*4) Testing capacities are shared between the regions.

We do not allow for migration between the nodes of the network, and individuals remain assigned to their initial region at all times. Transmissions across regions occur through temporary travel (and consequent interactions) of some individuals between regions. However, individuals continue to be assigned to their initial (home) region, as this assignation is crucial for the definition and description of the target transmission rates (Table 2).

**Table 2:** Parameters for all numerical scenarios

Economic parameters		Epidemiological parameters	
n	3	${ℐ}_{0}$	2.5
ρ	0.0	$\alpha_{L}$	1/15
$\bar{N}$	$\sum_{i}N_{i}\left(0\right)=1.0$	$\alpha_{D}$	1/15
GDP	50000[$∕c]∕365[d]=137.0[$∕c∕d]	$\theta_{LH}$	$1-{\left(1-0.0065∕0.15\right)}^{\alpha_{L}}=0.002948$
Ψ	$10^{6}\left[\$\right]∕GDP=7300\left[GDP\right]$	$\theta_{DH}$	$1-{\left(1-0.0065∕0.15\right)}^{\alpha_{D}}=0.002948$
ICU ‐ Cap	$\bar{N}\cdot 0.0003$	$\bar{\mu_{H,j}}$	0.15∕12=0.0125
$\bar{C_{M}^{i}}$	(2∕3⋅190$+1∕3⋅1330$)∕GDP=4.161[GDP]	$\bar{\alpha_{H,j}}$	0.85∕12=0.07083
$\bar{C_{V}^{i}}$	5$∕GDP=0.0365[GDP]		

### 5.1 Calibration

We assume, that within and across regions, there is an uncontrolled (baseline) transmission rate of $\beta_{i,j}$. Reducing the transmission rate through social distancing and lockdown measures is assumed to imply quadratic costs. The planer has the possibility to choose target values for all transmission channels $u_{i,j}$ separately. This means that not only can the transmission targets be chosen differently for interactions within and across different regions, but we explicitly allow for a distinction between the target rates $u_{j,k}$ and $u_{k,j}$. This enables us to model heterogeneous policy schemes, as were enacted by many countries during the pandemic. Notably, mandatory quarantine policies for incoming travelers have been frequently and repeatedly enacted by many countries over the course of the pandemic as a measure to curb the import of infections from other regions. While such measures have often been implemented to varying degrees, applying, for example, only to high‐infection regions, they have also been asymmetric in the following sense: Implementing preventive quarantine in region j (to return to our framework) for visitors from region k (and also people returning to j from k) reduces the transmission of infections of region k on region j. However, region k does not necessarily need to implement the mirror strategy, that is, to quarantine individuals traveling from j to k. Hence, the two transmission rates $u_{j,k}$ and $u_{k,j}$ can actually be targeted distinctly.

Trying to reduce transmission rates to zero would lead to a complete halt of all economic behavior, and the cost would then amount to the full GDP produced over this time period. The quadratic loss function then describes the increasing difficulty of transferring economic activity into contexts (e.g., home office) with lower risks of disease transmission and of closing down traffic across regions.

(18) $$C_{U}\left(u,t,i,j\right)={\left(1-\frac{u}{\beta_{i,j}}\right)}^{2}\cdot \bar{C_{u}^{i,j}}$$

The parameter $\bar{C_{u}^{i,j}}$ measures the GDP corresponding to activities that generate transmission of the virus from region i to region j. The GDP of all regions is normalized in units per day and person. Again we normalize the total GDP per day and person, which is the sum over all three regions, to 1, that is, $\sum_{i,j}\bar{C_{u}^{i,j}}=1$. Each region is assumed to produce one‐third of the total GDP (corresponding to its population size, see assumption (*G*1)), where 50% of each regional share results from interactions within the home region and 25% from interactions with each of the two other regions, that is,

(19) $$\bar{C_{u}^{i,j}}=\left\{\begin{array}{ll} N_{i}\left(0\right)\cdot 0.5\; & \;\text{for}\;\;i=j\\ N_{i}\left(0\right)\cdot 0.25\; & \;\text{for}\;\;i\neq j. \end{array}\right.$$

The region‐specific uncontrolled transmission rate is defined as follows:

(20) $$\beta_{i,j}=\left\{\begin{array}{ll} 0.5\cdot {ℐ}_{0}\cdot \alpha_{L}\; & \;\text{if}\;\;i=j\\ 0.25\cdot {ℐ}_{0}\cdot \alpha_{L}\; & \;\text{if}\;\;i\neq j. \end{array}\right.$$

Assuming that each light case (that does not escalate to a heavy case) infects an average number ${ℐ}_{0}$ of people, we can define the uncontrolled transmission rates by dividing through by the average time in the infectious stage $1∕\alpha_{L}$. Furthermore, transmissions have to be assigned to contacts with different groups. As we do for the economic costs, we assume that 50% of contacts take place within each region and 25% across the boundary with each of the two neighboring regions.^22^ A complete lockdown and elimination of all interaction is not plausible due to necessary activity, for example, in the medical sector or in the provision of essential goods and services. Thus, we assume that all transmission rates have a lower bound $\underline{u_{i,j}}$ which we set to 10% of the uncontrolled rates in our illustrations, that is, $\underline{u_{i,j}}=0.1\cdot \beta_{i,j}$.

The average time spent with light or no symptoms while being infectious is assumed to be 15 days for both undetected and detected cases, such that $\alpha_{L}=\alpha_{D}=1∕15$. We can infer the probability of escalation of a light case, that is, the risk of hospitalization, from the general infection fatality rate (IFR) and the mortality risk of patients in hospital treatment. Assuming an IFR of 0.0065 and a mortality risk of 15% for hospitalized cases, we obtain an escalation probability of 0.0065∕0.15=0.043. Spreading this probability over the average dwell time in the light case state, we obtain a per‐diem escalation rate of $\theta_{LH}=1-{\left(1-0.043\right)}^{\alpha_{L}}=0.002948$ (and an analogous definition for $\theta_{DH}$).^23^

Finally, we need to specify the cost functions. World Bank data shows that income within western and northern Europe mostly lies within 40,000–65,000$ per capita. Hence, we choose the statistical value of life to be 20 times the GDP per capita (per year) as in Alvarez et al. (2021). For hospital treatments and for tests we assume linear and identical costs for all regions,

$$\begin{array}{ccc} C_{M}\left(H_{j},t,j\right) & = & \bar{C_{M}^{j}}\cdot H_{j}\\ C_{V}\left(v_{j},t,j\right) & = & \bar{C_{V}^{j}}\cdot v_{j}. \end{array}$$

Since we normalized the total population to 1, the cost parameters $\bar{C_{M}^{j}}$ and $\bar{C_{V}^{i}}$ correspond to the treatment costs per day and person, as measured in units of per capita GDP per day. Heavy cases in our framework are not only ICU patients, but also patients admitted to the hospital with less intense treatment needs. Recent studies have found a wide range of costs per patient per day receiving ICU and general treatment. For our analysis, we take the values from Edoka et al. (2021) who found costs of 190$ (general ward) and 1330$ (ICU) per patient day. According to the US Center for Disease Control (CDC), about one‐third of hospitalized cases require intensive care. Consequently, we take the expected costs for a heavy case to be 443$ per patient day. The costs of testing vary strongly with the type of test. However, generally, the price is relatively low compared to all other costs (we set it to 5$). It is thus capacity that acts as a potentially limiting factor.

ICU capacity is assumed to be fixed at 30 beds per 100,000 individuals.^24^ Testing capacity is very low at the beginning of a pandemic, but increases over time. In our case, we assume that testing capacity increases from 10 up to 1000 tests per 100,000 individuals per day.

$$\bar{V}\left(t\right)=\bar{N}\cdot \left(0.0001+0.01\cdot \frac{t}{\bar{T}}\right).$$

The mortality and regeneration rates of hospitalized individuals are assumed to depend on the number of heavy cases. We assume that the sum of the two rates is constant and describes an average hospitalization time of 12 days. For an ICU with spare capacity the share of hospitalized people dying is assumed to be 45%. Translated to the total number of hospitalized patients, the base mortality is 15%, and thus related to the average time spent in the hospital according to $\bar{\mu_{H,j}}=0.15∕12$ and $\bar{\alpha_{H,j}}=0.85∕12$. As soon as the ICU capacity is exhausted (the expected number of ICU patients is $\bar{H}∕3$), we assume that any further patient with intensive care needs has a 100% mortality risk. Following the calculations of Caulkins et al. (2020), this implies that the average mortality for all heavy cases is increasing concave with the number of heavy cases approaching 1/12 in the limit. For computational reasons, we propose the following functional forms, which smooth the nondifferentiable point of $\mu_{H}\left(\bar{H},t,j\right)$, where $\bar{H}∕3$ reaches the ICU cap. Smoothing ranges from this point up to a 10% overload of the ICU capacities:

$$\begin{array}{ccl} \mu_{H}\left(\bar{H},t,j\right) & = & \left\{\begin{array}{ll} \bar{\mu_{H,j}}\; & \;\text{if}\;\;\frac{\bar{H}}{3}<\mathrm{ICU}\\ \bar{\mu_{H,j}}+\bar{\alpha_{H,j}}\cdot \left(1-\frac{\mathrm{ICU}}{\bar{H}∕3}\right)\cdot f\left(\bar{H}\right)\; & \;\text{if}\;\;\mathrm{ICU}\leq \frac{\bar{H}}{3}\leq 1.1\cdot \mathrm{ICU}\\ \bar{\mu_{H,j}}+\bar{\alpha_{H,j}}\cdot \left(1-\frac{\mathrm{ICU}}{\bar{H}∕3}\right)\; & \;\text{if}\;\;1.1\cdot \mathrm{ICU}\leq \frac{\bar{H}}{3} \end{array}\right.\\ \alpha_{H}\left(\bar{H},t,j\right) & = & \bar{\mu_{H,j}}+\bar{\alpha_{H,j}}-\mu_{H}\left(\bar{H},t,j\right). \end{array}$$

**Figure 2:**
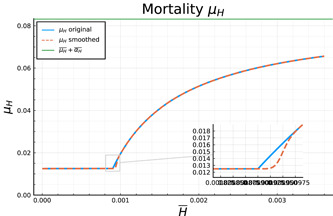
Original and final smoothed version of the mortality function describing congestion in the health sector.

The function f(⋅) is a sigmoid function of form $f\left(x\right)=\frac{x^{3}}{x^{3}+{\left(1-x\right)}^{3}}$ which is sufficient for a smooth final function. Figure 2 shows the general form of $\mu_{H}$ and highlights the marginal differences between the original function (following Caulkins et al., 2020) and our smoothed version.

Finally, we assume that the three regions differ (only) in the number of initial infections, reflecting the realistic setting in which the pandemic breaks out and spreads within a single region of origin before spreading into other regions. We thus propose that the pandemic has already progressed within the first region (group), where 10% of the population are already infected at the starting time of the model. For the second group, we assume an initial prevalence of infections at 1% of the population, while the third region enters with a zero prevalence at the beginning of the time horizon. In Table 2 we summarize all parameters discussed in this section.

### 5.2 Results

In the following sections, we study four cases representing a sequence of increasing capabilities of the social planer: (i) “Uncontrolled,” that is, the development of the epidemiological states if no intervening measures are taken. (ii) “No Testing,” where the social planer is (only) able to introduce lockdown measures and social distancing orders. (iii) “Ineffective testing,” where the social planer also allocates tests (subject to the capacity constraint) for the identification of light cases, but where in the absence of effective contact tracing, tests have to be allocated randomly across the nondiagnosed population, that is, where κ=1. (iv) “Effective testing,” as the optimal solution for a scenario where effective contact tracing allows tests to be (almost) perfectly targeted at light cases, that is, where κ=0.1. Table 3 summarizes the set‐up for the different scenarios. First, uncontrolled development is discussed in Section 5.2.1. The controlled cases are investigated in Sections 5.2.2–5.2.4.

**Table 3:** Qualitative comparison of the four different cases analysed

Case	Transmissions	Testing	κ
Uncontrolled	Uncontrolled	None	–
No testing	Optimally controlled	None	–
Ineffective testing	Optimally controlled	Optimal	1
Perfect testing	Optimally controlled	Optimal	0.1

#### 5.2.1 Uncontrolled development

**Figure 3:**
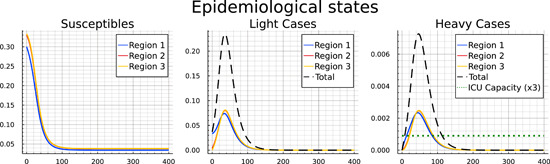
Pandemic development for the “Uncontrolled” case.

Figure 3 presents the most important epidemiological states, as described by Equations (1)–(4) for an uncontrolled pandemic: susceptibles, light cases, and heavy cases. The blue solid line denotes the corresponding state for region 1, the red one that for region 2 and the yellow one that for region 3. The dashed black line denotes the total number of light and heavy cases, respectively. In the absence of any controls the different initial conditions across the regions make hardly any difference for the course and outcome of the pandemic. The spike in light and heavy cases is only delayed by a few days for the second and third region. Furthermore, an equilibrium is reached after 150 days where there are practically no susceptibles nor light cases left which could trigger new infections (recall that diagnosed and heavy cases are assumed to be isolated). Hence, the pandemic is short but severe, as follows from the fact that ICU capacity, denoted by the dotted green line in the panel is exceeded by the total number of heavy cases (dashed black line) over almost the full course of the pandemic. This causes a high number of deaths, which, as we will see, could have been prevented by measures aimed at controlling the disease.

Table 4 includes a summary of the terminal outcomes of the pandemic after 400 days for each region as well as for all regions in total.^25^ The first four columns show the population shares (within each region and in total), who have not been infected (S), recovered from the disease ($R_{L}$ and $R_{D}$), and died (M), respectively. The final four columns show (from the left) the cumulated costs of the pandemic up until Day 100, 200, 300, and 400, respectively.^26^ As we see in Figure 3 the pandemic is raging mainly over the first 100 days, and has “burnt out” by Day 200. Thus, costs do not change afterward. The table also mirrors that there is little difference across regions. Without any control measures the disease can quickly spill over from region 1 to region 2 and 3 and take the same course afterward.

**Table 4:** Summary and comparison of the endstates of the pandemic and the costs of the pandemic across the three different regions for the four scenarios analysed

	Epidemiological states at t=400				Costs until t (in GDP p.c.p.d.)			
	S (%)	$R_{L}$ (%)	$R_{D}$ (%)	M (%)	*t* = 100	*t* = 200	*t* = 300	*t* = 400
Uncontr. case								
Region 1	10.29	85.91	0.87	2.93	70.23	71.91	71.92	71.92
Region 2	11.39	84.86	0.81	2.94	70.31	72.23	72.24	72.24
Region 3	11.51	84.74	0.8	2.95	70.32	72.27	72.28	72.28
Total	11.06	85.17	0.83	2.94	210.86	216.41	216.44	216.44
No testing								
Region 1	27.58	69.29	2.6	0.46	19.92	27.22	30.26	31.52
Region 2	40.03	57.34	2.14	0.38	7.18	15.74	20.6	22.58
Region 3	41.5	55.93	2.09	0.37	5.34	13.69	18.76	20.83
Total	36.37	60.85	2.28	0.41	32.45	56.65	69.63	74.93
Ineffect. testing								
Region 1	31.31	63.99	4.22	0.44	19.25	25.39	28.17	29.12
Region 2	44.14	48.92	6.53	0.36	7.06	14.92	18.93	20.3
Region 3	45.57	47.36	6.66	0.35	5.35	13.17	17.32	18.74
Total	40.34	53.42	5.80	0.38	31.66	53.49	64.42	68.16
Effective testing								
Region 1	54.02	35.62	10.07	0.29	15.07	18.25	18.8	18.82
Region 2	68.06	21.8	9.94	0.2	6.18	10.12	10.81	10.84
Region 3	69.24	20.98	9.59	0.2	5.31	9.32	10.02	10.05
Total	63.77	26.13	9.86	0.23	26.56	37.69	39.63	39.7

*Note*: Endstates are given in percentage of the initial population size of each region, resp. the total population. Costs are given in units of GDP per capita per day (GDP p.c.p.d.).

Interestingly, in region 1 slightly fewer people die as compared to regions 2 and 3, which is partly due to a first‐mover advantage in the ICU. The fact that the early‐coming heavy cases from region 1 are hospitalized in a situation without congestion yet gives them the advantage that all (or many) of them receive the required care. A short time later, the ICU is congested and the available capacity is divided across the three regions, leaving many heavy cases unserved in all regions.^27^

Turning to infections, we see that in each region only slightly more than 10% of the population remain susceptible and more than 85% are either recovered light or heavy cases. Nearly 3% of the population have died during the pandemic, predominantly as a result of an overwhelmed health sector (recall the right panel in Figure 3).

#### 5.2.2 Controlled development

**Figure 4:**
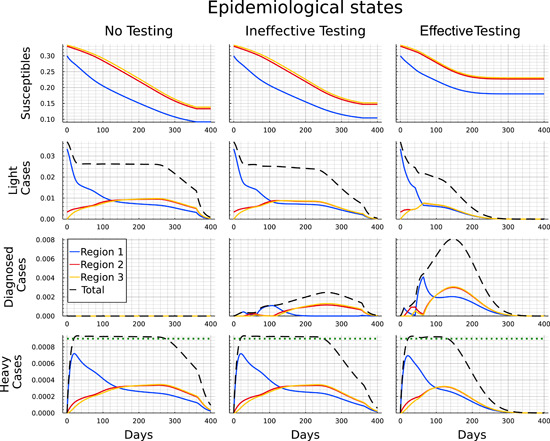
Comparison of the pandemic development for the “No testing,” “Ineffective testing,” and “Effective testing” case.

In this section, we compare the development across a sequence of three scenarios in which the social planner is assumed to have increasing capabilities of controlling the disease. Figure 4 allows for a comparison of the epidemiological development of the pandemic over time between the no‐testing, ineffective‐testing, and effective‐testing cases.

The first column illustrates the development for each of the regions (colored lines) and in total (black dashed line) when tests are not available. Three key distinctions characterize the controlled scenarios from the uncontrolled scenario. First, the duration of the pandemic is much longer and basically stretches until a vaccine becomes universally available at the end of the time horizon. Second, the main initial focus of the measures lies on pushing down infections in region 1 to a level at which the number of light cases is as low as in the other regions. After that (i.e., after ≈130 days) infections in region 1 start to lie below those in the other regions, which is due to the smaller number of susceptibles, following the initial period. As one would expect, heavy cases follow the course of the light cases with a delay. Third, the ICU capacity constraint is surpassed only slightly,^28^ but again for a long time (between Days 20–270). These features correspond to “classical” curve‐flattening as an optimal policy to contain the number of deaths by avoiding excessive ICU congestion while at the same time containing the costs of shutting down the economy. Such a policy remains in place up until either herd immunity is reached (which lies well beyond the time horizon for the COVID‐19‐like disease underlying our calibration) or up until the vaccine is universally available. The second part of Table 4 summarizes the outcomes for this scenario after 400 days. It can be seen that the impact of the pandemic on fatalities is significantly reduced. The death rate drops from nearly 3% of the population to values between 0.37% and 0.46%. Furthermore, we see that the direct lockdown measures lead to a decrease in the number of infected individuals in the first region, while in the second and third one the spreading of the disease is significantly slower. After the end of the pandemic, around 36% of the total population across all regions have not been infected.

By slowing down transmissions and thereby reducing the number of light cases, the lockdown measures bring down the number of heavy cases from around 3.8% of the population to around 2.7%.^29^ As it turns out, this is tantamount to containing the number of heavy cases to a level below or slightly above the capacity limit. This confirms that the optimal strategy for lockdown measures is to keep the number of heavy cases at a level, that does not overburden the ICU facilities, but at the same time contains the (economic) costs of lockdown.

We can now use the second column of Figure 4 to identify the impact of the availability of tests, which are subject to a capacity constraint. For this scenario we assume there is no infrastructure for contact tracing and the disease hardly shows specific symptoms for light developments of the infection. Consequently, tests need to be allocated randomly across all members of the population without a diagnosis. The main epidemiological development in the case with ineffective testing looks qualitatively similar to the case without testing. However, the number of light cases continues to decrease mildly between Days 20 and 250 instead of remaining at a plateau. While this mostly corresponds to the number of diagnosed cases turning positive, the number of never‐infected individuals (i.e., the susceptibles at t=400) is also slightly higher in all three regions. Furthermore, the ICU capacities are used to the full extent about 20 days less as the total number of heavy cases drops slightly earlier.

Finally, we present the optimally controlled epidemiological development when testing is effective in the sense of being better targeted at the group of light cases, a setting that is broadly consistent with efficient contact tracing. In this scenario, we obtain significant differences to the two previous cases of “no testing” and “ineffective testing,” with the availability of testing now showing noticeable impacts.

In the third column in Figure 4 we observe that the pandemic ends after approximately 250 days and, thus, before the arrival of universal vaccination after 360 days. This is indicated by the number of light cases being close to zero and the number of susceptibles showing no significant change any more. During the first 60 days the development appears to not significantly differ from the development with ineffective testing.^30^ Subsequently, however, we observe a steeper decline in the number of light cases in region 1. And although the light cases reach a similar peak in regions 2 and 3 regardless of the effectiveness of testing, in the case of effective testing the numbers immediately drop from Day 60 onward, while they stay on a plateau for roughly 150 days if testing is ineffective.

The decreasing number of light cases in case of effective testing also corresponds to the strongly increasing number of detected cases. The peak is earlier and nearly threefold the peak under ineffective testing. Note that the number of diagnosed individuals is higher throughout under effective testing even though the number of light cases is significantly lower. Hence the increased effectiveness clearly overcompensates the smaller pool of undetected light cases. The ICU capacities are also in full usage in case of effective testing. However, as the infection dynamics have been slowed down, the number of heavy cases drops below this threshold about 120 days earlier than in the other controlled cases.

Comparing the end results of the epidemiological development after 400 days as shown in the second half of Table 4 we see that the impacts of testing strongly vary with respect to its effectiveness. For ineffective testing, an additional 4% of the total population were protected from getting infected during the pandemic (an increase from 36.37% to 40.34%), while the total number of deaths only dropped from 0.41% to 0.38%. In the case of effective testing, however, only slightly more than a third of the population got infected, and the number of deaths was reduced to 0.23%, just a little more than half the number without testing. This is largely owing to ICUs operating at capacity limit over a much shortened time span of only about 130 instead of 250 days.

#### 5.2.3 Optimal transmission rates and testing

**Figure 5:**
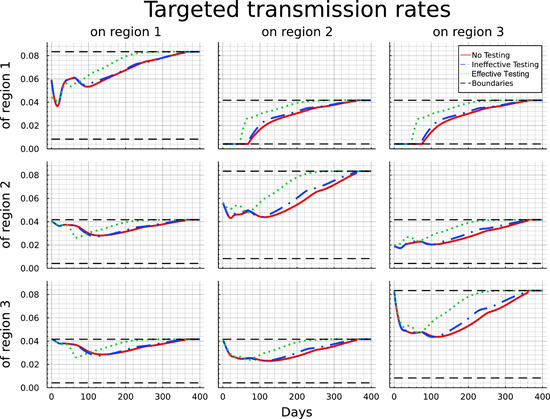
Optimal target transmission rates for the “No testing,” “Ineffective testing,” and “Effective testing” case.

In this section, we discuss the optimal strategies of the social planer for transmission rates and testing in the various scenarios. In Figure 5 we present the optimal target transmission rates for the different scenarios. The upper boundary (black dashed line) corresponds to the transmission rates in the case of an uncontrolled pandemic. The red solid lines describe the optimal transmission rates if tests are unavailable, the blue dash‐dotted lines for the case of ineffective testing and the green dotted lines for effective testing. We first focus on the target transmission rates when testing is not an option for the social planer.

Let us begin with the within‐region measures, corresponding to the panels along the diagonal from top‐left to bottom‐right. Within region 1, the relatively high initial level of infections is curbed by the introduction of a short lockdown (a type of wave breaker). After some relaxation, measures are tightened again to control infections that now tend to be imported from regions 2 and 3. From Day 100 onward restrictions are gradually lifted until vaccination is available after 360 days. For region 2, the pattern of internal lockdown is qualitatively similar. Here, the initial tightening of the lockdown is in line with transmissions from imported cases. Notably, while the lockdown is softer than the one in region 1 initially, this reverses from Day 50 onward, where restrictions within region 2 are relaxed more cautiously and tend to remain in place over a longer period. The pattern of the internal lockdown within region 3 is similar to region 2 with the only difference being its more gradual introduction at the beginning of the planning horizon, following from the absence of internal infections at the starting point. Overall, the pattern suggests that the planner has an incentive (i) to equalize the pattern of the pandemic and its control across the three regions (which follows from the minimization of convex control costs), and (ii) to smooth out the lockdown over time. While this is relatively successful within the “follower” regions 2 and 3, the initial presence of the disease makes this objective more difficult to achieve within region 1.

Turning to cross‐region measures, initial observation is that for all regions the restrictions are considerably stronger than the internal measures. This suggests that the planner is much more ready to impose travel bans, which is particular true in respect to region 1, where transmissions into the other two regions are initially reduced to their lowest possible level, implying the strongest feasible measures. Similar yet more modest restrictions are implemented to avoid the spillover of transmissions from region 2 into region 3, which initially features no infections.

Conversely, and assuming the possibility of asymmetric restrictions, measures taken at constraining transmissions from regions 2 and 3 into region 1 and from region 3 into region 2, respectively, are more modest and implemented only gradually in line with the scope for “reimporting” infections. This asymmetric behavior corresponds with the implementation of a mandatory quarantine (or similar measures to ensure noninfectiousness) for individuals of region 1 trying to travel to regions 2 or 3, while travel in the reverse direction can be done “without” precautionary measures. The same implementation of mandatory quarantine would hold for individuals returning to regions 2 or 3 after having traveled to region 1.

Referring back to Proposition 1 this asymmetric behavior becomes less surprising. Considering a special case of Proposition 1 with $k_{1}=j_{1}=k$ and $k_{2}=j_{2}=j$, a small rearrangement of the terms leads to the following equation:

(21) $$\frac{\partial C_{U}\left(u_{k,k},t,k,k\right)}{\partial u_{k,k}}\cdot \frac{\partial C_{U}\left(u_{j,j},t,j,j\right)}{\partial u_{j,j}}=\frac{\partial C_{U}\left(u_{k,j},t,k,j\right)}{\partial u_{k,j}}\cdot \frac{\partial C_{U}\left(u_{j,k},t,j,k\right)}{\partial u_{j,k}}.$$

Equation (21) connects all potential interactions between two nodes and illustrates that the product of marginal costs of transmission reduction *within* the two nodes has to equal the product of marginal costs *between* the two nodes. In our numerical example, given the similar strategies within each region and significantly stronger transmission reductions from region 1 to regions 2 and 3, Equation (21) directly implies reduced measures regarding transmissions in the opposite direction.

To explore the extent to which asymmetric measures improve the efficiency of disease control we additionally looked at a setting with symmetric target transmission rates, that is, where the planer is constrained to set the same transmission targets $u_{j,j}$ within regions j=1,2,3 and $u_{j,k}=u_{k,j}$ for all k and j across regions. The results, which are not presented here, indicate that (i) again the measures are chosen in a flattening‐the‐curve fashion, leading (ii) to a very similar level and distribution of mortality (and, thus, loss of lives). However, the economic costs of controlling the disease (see Section 5.2.4 for greater detail) are about 14% higher under symmetric measures and total cost are about 9% higher.

The introduction of ineffective testing enables the social planer to modestly reduce the lockdown measures and allow slightly higher transmission rates at certain points in time (blue dash‐dotted lines). While the optimal target transmission profile within region 1 and the transmissions of regions 2 and 3 do not differ significantly, we can observe slightly reduced lockdown measures within regions 2 and 3 after testing capacities have been built up. Furthermore, the social planer allows for higher transmissions rates between regions 2 and 3 (in both directions) as well as from region 1 to regions 2 and 3.

To assess the potential impact of the testing capacity constraint we conducted a robustness check allowing for the maximum capacity of 1000 tests per 100,000 individuals right from the beginning. The results show that the additional tests are used exclusively in the first group and allow for a faster reduction of unidentified light cases. This “advance” against the disease is mainly used to relax lockdown measures and thereby to reduce the economic costs over the whole time‐horizon by about a quarter. The epidemiological development remains largely unaffected, and there is hardly any change in the usage of ICU capacity or total lives lost.

The impact of testing becomes remarkably more pronounced if testing becomes more effective (green dotted line). While the patterns are qualitative similar to the “ineffective testing” case the measures are more lenient and confined to a shorter time period. After 100 days all transmission rates within the network are significantly higher and in particular, there is no need for policy interventions any more after 250 days, as is evident from the lack of any significant difference between the target and the uncontrolled transmission rates.

**Figure 6:**
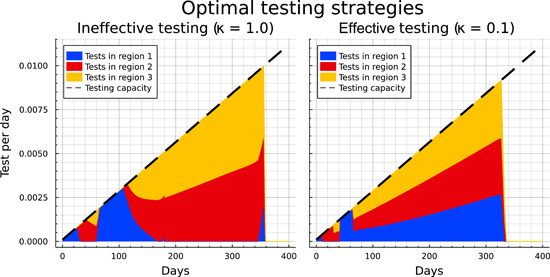
Optimal testing strategy in the “ineffective” and “effective” testing case.

Interestingly, however, efforts of protecting individuals in region 1 (from transmissions within group 1 and from the other two regions) are more intense between Days 40 and 80 under effective testing when compared to the other two controlled scenarios. Turning back to Figure 4 these efforts coincide with the peak in the light case numbers in region 2 and 3 and with a change in the test allocation, as we will discuss further down below in Figure 6. Finally, we note that even under effective testing a flattening‐the‐curve policy tends to be the optimal response to the pandemic peak.

Figure 6 illustrates the optimal allocation of the available tests across the three regions over time. The left panel shows the strategy for ineffective testing, the right for effective testing. Testing capacities are used to their full extent (i.e., 9 is binding for all t) at almost all times, reflecting that testing is relatively cheap in comparison to all other measures and potential costs. Figure 6 shows that a distinct allocation pattern across the three regions is present for both levels of testing effectiveness. Testing starts exclusively in region 1, the initial hotspot, for a little more than 20 days (about a week for effective testing, respectively). The focus subsequently shifts to region 2, followed by a mixed allocation across regions 2 and 3, before between Days 75 and 100 (respectively Days 60 and 80 for effective testing) testing effort is again concentrated on region 1. This circular pattern is also visible in the pattern of diagnosed cases (see Figure 4) and broken only from Day 100 (respectively Day 80 for effective testing) onward, where the number of infected cases has about equalized across the three regions. Despite the qualitative similarities, effective testing results in the shortening of the time span of each part of the pattern.

From then on the patterns of ineffective and effective testing evolve differently. Ineffective testing is gradually shifted away from region 1 and shared between regions 2 and 3 which feature a higher number of light cases (due to the later epidemiological peak in these regions). Between Days 180 and 340 tests are shared to roughly equal amounts between regions 2 and 3, while there is a small spike in tests in region 1 shortly before a vaccine becomes available at the end of the time horizon. For effective testing the brief period of focused region 1 testing is followed at Day 80 by a switch toward an equal distribution of tests across the regions for the remaining duration of the pandemic.

Recall from Figure 4 that under effective testing the number of undetected light cases is very low from Day 250 onward. Although the disease is under control, the planner continues to use tests up until Day 340 in a precautionary and low‐cost way to suppress any potential resurgence. Notably, testing is abandoned altogether for the last 20 days before the anticipated arrival of the vaccine at Day 360.

#### 5.2.4 Cost composition

In a last step we assess impacts of the different scenarios on the aggregated social costs of the pandemic. Unsurprisingly, the costs arising under the uncontrolled development of the pandemic are significantly higher than in any of the controlled scenarios. The right part of Table 4 summarizes the cumulative costs for the three regions and in total at different points in time (100, 200, 300, and 400 days, respectively). In the uncontrolled case the costs are nearly equally distributed across the three regions and are incurred almost entirely during the first 100 days of the pandemic. This corresponds to the number of heavy cases drastically overburdening the ICU capacities leading to a high number of fatalities. As there are no lockdown measures or testing efforts, all costs relate to hospital treatment expenditures and the value of lives lost. Treatment costs play only a marginal role at less than 1% of total costs.^31^ To contextualize the total costs of 216 units of GDP per capita per day, recall that the population size is normalized to one. Hence letting the pandemic spread through the population in an uncontrolled way implies that the value of lives lost during the first 100 days amounts to roughly two‐thirds of the total yearly GDP.

The remaining results in Table 4 show that controlling the disease yields drastic reductions in the total (health and economic) cost of the pandemic. Allowing for reductions in the transmission rates brings the costs down by some 65% to 74.93 units of GDP per capita per day. Another 3% cost reduction compared to the uncontrolled case is possible through the introduction of ineffective testing. Again more significant improvements can be achieved if testing is effective. The total cost of 39.7 units of GDP per capita per day are close to half of the costs in the absence of testing and represent a mere 18% of the costs associated with an uncontrolled spread of the pandemic.

Controlling the disease also bears on the distribution of costs across time and regions. Region 1, the original hotspot of the pandemic, is responsible for a significantly higher share of costs as compared to regions 2 and 3, which end up with more similar levels of aggregated costs. For all regions the costs are spread more smoothly over time, reflecting the curve‐flattening strategy.

**Figure 7:**
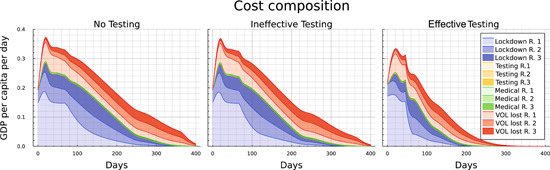
Origins of costs for the “No testing” (left), “Ineffective testing” (middle) and “Effective testing” case (right).

A more distinct analysis of the composition of health and economic costs can be deduced from Figure 7. This figure presents the costs profiles of the three controlled scenarios and allows for a comparison between them. Furthermore the costs are composed into their different sources and attributed to the regions, where they arise. Noting that the costs for testing (yellow) and medical treatments (green) are insignificant in all scenarios, we will focus on the two main driving factors: lockdown measures and value of lives lost. In all three controlled cases it is optimal to incur high lockdown costs to reduce the number of fatalities (and associated losses in value of life) especially within the first 40 days. The most significant part of lockdown costs results from transmission reductions in relation to region 1. As the pandemic develops over time, the costs associated with lockdown measures and fatalities become more balanced. Toward the end of the pandemic, when transmission rates return close to their uncontrolled values, the value of lives lost constitutes the majority of costs albeit at low levels. While costs associated with lives lost shift from region 1 toward regions 2 and 3 over time, the aggregated absolute value remains fairly constant (again reflecting the “flattening‐the‐curve”‐strategy).

The availability of imperfect testing lowers the cost of lives lost by some 6%, the economic cost of lockdown by some 11%, and total costs by some 9% relative to the “No testing case,” but affects only marginally the overall cost profile. Testing becoming highly effective not only leads to the cost of lost lives being cut by some 51% and the economic cost by some 44%, but also implies a significant shift in the cost profile. While the cost structure is fairly similar to the other two controlled cases up until a comparable peak at Day 60, costs drop more substantially under effective testing afterward. Effective testing also allows for a more even distribution of costs across and within the three regions. Notably, while the economic costs associated with lockdown measures are eliminated after roughly 200 instead of 300 days, the costs associated with the loss of lives have also been reduced to a negligible level by Day 300 in the “effective testing” case. This is in contrast to the “ineffective testing” case, where deaths accrue up to and beyond Day 360.

#### 5.2.5 Summary comparisons

We conclude the analysis by comparing the four (main) scenarios on the basis of a number of key outcomes, as summarized in Table 5.

**Table 5:** Comparison of the four (main) scenarios

		Uncontrolled	No testing	Ineffective testing	Effective testing
Endstates (*t* = 400)	S	11.06%	36.37%	40.34%	63.77%
	$R_{L}$	85.17%	60.85%	53.42%	26.13%
	$R_{D}$	0.83%	2.28%	5.80%	9.86%
	M	2.94%	0.41%	0.38%	0.23%
Agg. costs in GDP p.c.p.d. (*t* = 400)	Total	216.44	74.93	68.16	39.7
	VOL lost	214.56	29.6	27.79	16.85
	Lockdown	0.0	44.0	39.04	22.03
End of spread (days)		161.0	360.0	360.0	286.0
End of full ICU‐usage (days)		113.0	274.0	250.0	132.0
End of lockdown measures (days)		0.0	360.0	360.0	249.0

The first part of the table allows for an immediate comparison across the pandemic endstates in terms of the (remaining) susceptible population, the diagnosed and undiagnosed recovered cases, as well as the deceased at time t=400. The second part summarizes the total costs after t=400 and the value of lost lives and lockdown costs as the two main contributing factors (aggregated over all regions). We leave these entries for ease of comparison but without further comment.

The last three rows record a number of important dates in the progress of the pandemic. “End of Spread” defines the point in time at which the total number of susceptibles changes by less than 1 in 10,000, which is tantamount to a standstill of the pandemic. Thereby, we recall that in the “Uncontrolled” case the pandemic is over very quickly after 161 days but at enormous costs in terms of lost lives. In contrast, for both the “No testing” and “Ineffective testing” cases, the end of the spread does not occur before a vaccine is available. In contrast, “effective testing” combined with an optimal lockdown policy allows to shorten the pandemic by 74 days.

The second row shows the first day at which the ICU is no longer at (or beyond) its capacity limit. Again in the uncontrolled case this happens early on, as the disease rages heavily within the first 3 months. This hides, of course, the number of heavy cases not receiving treatment and dying due to overload. More notably, we see that testing, regardless of its effectiveness, allows for an earlier relief of the strain on the ICU. In the limit, “effective testing” more than halves the time span of congestion, whereas in case of “ineffective testing” the reduction is more minor.

The last row shows the date at which all lockdown measures are lifted, as defined by the time at which all target transmission rates are within 1% of the uncontrolled rates. Again “effective testing” is a powerful instrument in bringing down to 249 the number of days over which lockdown measures of some intensity are implemented. “Ineffective testing” leads to no reduction in the duration of the lockdown (of some intensity) below the full 360 days up until the availability of universal vaccination.

## 6 CONCLUSIONS

This paper considers an extended SIR model across a network. It combines several important new features that are relevant for the COVID‐19 pandemic but generalize to other infectious diseases that involve severe illness. The model distinguishes light and heavy courses of the disease, where the latter are characterized by the need for hospital or ICU treatment. Whereas a heavy case is per definition known and excluded from infectious contacts, light cases need to be detected by testing efforts. We allow for parametric variations in the extent to which tests can be targeted effectively, for example, due to high capacity of contact tracing. This influences the success rate of detecting people with a light course by testing efforts. Detected people suffering a light course are quarantined and do not contribute further to new infections. We consider that ICU capacity is constrained. If the capacity constraint is exceeded people with a heavy course cannot be treated in the ICU and die.

We study this model on a network, where the population at each node is subdivided into the relevant compartments. The network structure of the model can represent different geographical regions, a case we consider in the main body of the article; different subgroups of the population, a case which is available in the Supporting Information material in the Online Appendix; or, indeed, both. Disease transmission occurs both within and across network nodes. By the optimal choice of a set of target transmission rates (within certain bounds) the decision‐maker can reduce the spread of the disease. Depending on the model interpretation it is possible that the ICU capacity corresponds to a single node or is shared between (some of the) nodes.

In general we show that for an interior solution with respect to the target transmission rates the optimal allocation equalizes the marginal rate of transformation across the network with respect to both containing the spread of the disease and protecting from it. Regarding the containment of spread we also find that the marginal rate of transformation depends only on the current shares of light cases in the nonisolated population in the two respective regions. Furthermore we find that the relative size of the testing pool is one decisive factor for the relative allocations of testing and lockdown measures. By reducing the size of the testing pool, more effective testing can thus be shown to lead to a shift in the allocation from containment measures, such as lockdown or quarantining, to testing.

Studying the optimal allocation of target transmission rates (i.e., optimal containment) and testing strategies for a numerical example in which the pandemic spreads from one region into two otherwise identical regions, we can summarize the following key messages.

1. The analysis demonstrates that containment by way of lockdown and quarantining measures is effective in terms of saving lives and reducing total costs. While this depends on our assumptions about the value of a life saved, the cost reduction is so large as to make our finding robust. This generalizes earlier findings to a network setting.
2. For our calibration, the optimal containment policy is geared toward “flattening‐the‐curve” and thereby containing the number of heavy cases to a level that ICU capacities are (just) able to cope without significant congestion.
3. Lockdown within regions is typically weaker than measures undertaken to contain the transmission of the pandemic across regions. Initially, strong measures are taken to contain the disease within the region of the initial outbreak but these are adjusted over time to establish a course of the disease that is symmetric across the three regions. The common practice followed by many countries during the pandemic of imposing travel bans provoked by spikes in infections in foreign countries finds some support in our model.
4. Testing, on which countries have relied to very different extent during the COVID‐19 pandemic, proves to be effective by relaxing the trade‐off between saving lives and containing economic costs. However, the extent to which this is true hinges on (i) the effectiveness of contact tracing as a necessary requirement for tests that are targeted at (likely) light infections; and (ii) on limits to the testing capacity. If tests are in limited supply (especially during the early stages of the pandemic) only well targeted testing has the potential to speed up the termination of the disease and, thereby, to cut the time over which lockdown measures are required. In policy terms, this requires the availability of effective tracing capacities. The massive cost reductions over the pandemic afforded by the introduction of effective (rather than ineffective) testing indicates that policy‐makers should be willing to invest significantly into improvements in testing efficacy.
5. Both lockdown and testing are initially concentrated on the hotspot(s) with high numbers of current infections with the twin aim of flattening‐the‐curve and containing spill‐over effects into other regions. Policies are subsequently adjusted in a way that they become more similar over time.
6. When the policy objective is the minimization of the total cost of the pandemic across all regions, then both lockdown and testing policies are chosen in a way that aims at equalizing the pandemic across regions. While this leads to the assimilation of policies across regions, such an endeavor is the more successful the more targeted testing can be used.

Our model features a number of limitations, most notably that it is set out from a social planer perspective. This provides a characterization of the first‐best allocation as a yardstick and—in its regional interpretation—reflects the policy that should be implemented by a strong centralized government. But it does not yet capture well the policy outcomes in a decentralized setting where (i) local decision‐makers at each node of the network—in the regional context best thought of as regional authorities— optimize a local (regional) objective but not social welfare; and/or (ii) where individuals are taking voluntary decision in respect to self‐protection. While the modeling in (ii) is difficult to integrate with the planer model, one natural extension to the present model would be the consideration of a game between local decision‐makers. Comparison against the first‐best allocation would allow to identify inefficiency related to externalities across network nodes (regions) as well as possible coordinating measures on the part of the central government aimed at improving the allocation. Finally, we believe our framework to be flexible enough to be applied to a broad range of pandemic, population and economic settings, including consideration of social rather than regional networks and a variety of settings that involve asymmetric networks. We relegate these issues to future work.

## Supporting information

Supplementary Information

## Data Availability

Data sharing is not applicable to this article as no datasets were generated or analysed as part of the current study.

## Footnotes

1
https://covid19.who.int/, last access May 30, 2022.

2 Note that while our analysis is motivated by the ongoing COVID‐19 pandemic (including the calibration of the numerical analysis), our model and the epidemic, economic and demographic structures described as well as the policy mechanisms generalize to many other forms of present‐day epidemics. Thus while some of the insights on optimal testing and lockdown policies may come too late in the fight against COVID‐19, the insights are worth consideration for future pandemic events.

3 For example, Germany: 33.9, Italy: 8.6 intensive care beds per 100,000 people (year 2020). See de.statista.com for details.

4 For example, higher risk for a heart attack or a stroke, reduced lung capacity, taste disorder.

5 We chose the epidemiological parameters of the disease such as transmission, hospitalization, mortality, and recovery rates to reflect those of COVID‐19 found in the literature (see Section 5.1 for information on the calibration). However, we do not seek to replicate all aspects of what continues to be an emerging condition but give insight into optimal epidemiological strategies for potential new infectious diseases in the future, which pose risks similar to COVID‐19.

6 SIR models have been used to study infection dynamics for almost 100 years starting with the seminal paper by Kermack and McKendrick (1927). The enormous impact of the COVID‐19 pandemic has lead to a large number of recent papers using this type of models to analyze different aspects of the crisis. For a general and broad literature review we refer to Bloom et al. (2022).

7 To shorten the notation we will often use the expression (network) node j∈Ω for referring to a specific group our region.

8 As we are considering testing effort as a control variable, it is necessary to distinguish recovered individuals according to whether or not their infection was detected. Recovered that were detected (through testing or through a heavy course of disease) are assumed to be immune (at least for several months) implying that they are exempt from further testing. In contrast, recovered individuals that remained undetected cannot be distinguished from susceptibles, implying that they will be tested with the same effort.

9 The ongoing development of the COVID‐19 pandemic has shown that loss of immunity (obtained through infection or vaccination) over time significantly contributes to the infection dynamics. As this loss of immunity can have multiple reasons like the diminishing efficacy of vaccinations or the emergence of new mutations of the virus, significant modeling effort is required to adequately reflect this aspect of the COVID‐19 pandemic. While we are addressing some of these issues in upcoming work, we rather focus the present analysis on the network effects within a more standard SIR model without possibilities for reinfection.

10 Note that in general we can assume Ω to be a continuous space. The formulation of the dynamics and the derivation of the optimality conditions are quite similar. Then the resulting model is a distributed parameter optimal control model.

11 Note that we abstract from flows between different nodes, i.e., we do not allow migration between the different regional or social groups within the network.

12 We are not specifying whether a given target for the transmission rate is achieved through changes in the contact or infection rate, however, we assume, that the more cost‐effective measures are applied first, what also motivates the nonlinear costs.

13 This is reflected in many policies being justified by curbing the reproductive number to a value below one, and/or being conditioned on the rate of new infections.

14 Note that the other groups at each node $D_{j},H_{j},R_{j}^{D},M_{j}$ were isolated and diagnosed at some point during their infection and are hence not part of the population relevant for testing.

15 Note that $v_{j}\left(t\right)$ must be bounded, as otherwise the state $L_{j}\left(t\right)$ could turn negative. We assure that this is ruled out by assuming a constraint on the total number of performed tests (see (9)), reflecting that test kits including protective gear and, especially, laboratory capacity have been a scarce resource in particular at the beginning of the pandemic. This is also in line with the fact that in all countries the number of performed tests per day are below a maximum number of 5% of the total population (see de.statista.com).

16 The control constraints can be altered to account for different properties of the network. If, for instance, the network describes different regions, then each region may face an individual constraint, that is, $v_{j}\left(t\right)\leq \bar{V_{j}}\left(t\right)$ for j∈Ω.

17 Note that the terminal time is typically unknown to the policy‐maker. However, there is no loss in generality as T can always be chosen large enough for the policy‐maker to be certain beyond a margin of reasonable doubt that the pandemic would have ended through the availability of a vaccine or otherwise. In the case of COVID‐19 for instance, which in most industrialized economies emerged from March 2020 onward, policy‐makers were expecting as early as spring 2020 that vaccines would be available by spring 2021.

18 We assume that all susceptibles in the network are vaccinated within a very short spell of time. The real situation (at beginning of 2021), however, teaches us that vaccination is not an easy task and that its rollout may take considerable time. This lies outside of the scope and focus of our model and is investigated in, for example, Buratto et al. (2021). See also footnote 9.

19 For the purpose of this analysis, we assume that the vaccination delivers sterile immunity for all individuals, that is, the remaining undetected light cases cannot infect anybody after the vaccine becomes available. The COVID‐19 pandemic has shown that while vaccinations are not always able to eliminate the risk of infection completely, they are still very effective in reducing the number of hospital cases and case mortality. As we will see later in the numerical results, the treatment costs for infected individuals are miniscule compared to the costs for lockdowns and lives lost. Our assumption of sterile immunity does not significantly affect the salvage function then, since significantly reduced numbers of hospitalizations are unlikely to threaten ICU‐capacities and reduced mortality implies a very low total value of lives lost after the vaccine becomes available.

20 The presented optimality conditions hold for interior solutions. We omit the explicit presentation of boundary solutions, which is straightforward. The general formulation including boundary controls can be found in the Supporting Information: Online Appendix.

21 Recall that an increase in the target transition rate lowers costs, that is, that $\frac{\partial C_{U}\left(u_{k,j}\left(t\right),t,k,j\right)}{\partial u_{k,j}\left(t\right)}<0$.

22 We are aware that we are using a heuristic parameter ${ℐ}_{0}$ for the calibration of the uncontrolled transmission rates instead of the basic reproduction number ${ℛ}_{0}$. We chose this strategy for two reasons. First, the derivation of ${ℛ}_{0}$ in a multigroup extended SIR‐framework is quite involved (see Van den Driessche & Watmough, 2002, for details). Second, actual estimates of ${ℛ}_{0}$ for COVID‐19 cover a relatively broad range, so the ${ℛ}_{0}$ in our model resulting from the heuristic definition of the transmission rates is likely to be within this range. At the same time, the calibration of $\beta_{j,k}$ is significantly easier.

23 We adopted these parameter values from Caulkins et al. (2020, 2021).

24 Countries that are equipped approximately with that amount of ICU capacity are Germany 33.9 (year 2017), Austria 28.9 (year 2018) and US 25.8 (year 2018). See de.statista.com, for details.

25 An extended version of this table can be found in the Supporting Information: Online Appendix.

26 A decomposition of the costs with respect to medical costs and lost lives can be found in the Supporting Information: Online Appendix.

27 We have verified the first‐mover advantage by an additional numerical run without ICU capacity, where it turns out that the number of deaths is highest in region 1.

28 The fact that ICU capacity is surpassed at all might come as a surprise. Recalling, however, the smoothed mortality function in Figure 2, it becomes apparent that the first few cases that surpass the ICU capacity are subject to only a marginal increase in their mortality risk.

29 To obtain these figures add the (total) shares of $R_{D}$ and M from Table 4, while noting that all heavy cases and only heavy cases are either diagnosed survivors or deceased.

30 It appears that the low testing capacity is dominating the positive impact from higher testing effectiveness and the epidemiological development is only marginally different.

31 For a detailed table see the Supporting Information: Online Appendix.
