# Supplementary material for: Chasing up and locking down the virus: Optimal pandemic interventions within a network
Source: J Public Econ Theory. 2022 Jun 29:10.1111/jpet.12604. Online ahead of print. doi: 10.1111/jpet.12604 (PMC9350112; doi:10.1111/jpet.12604)
Supplement: Supplementary file 1 — Supplementary Information [file JPET-9999-0-s001.pdf]

Chasing up and locking down the virus: Optimal pandemic interventions within a network - Online-Appendix

Michael Freiberger[1*], Dieter Grass[1*], Michael Kuhn[1*], Andrea Seidl[2*] and Stefan Wrzaczek[1*]

[1*]International Institute for Applied Systems Analysis (IIASA), Schlossplatz 1, Laxenburg, 2361, Lower Austria, Austria.
[2*]University of Vienna, Oskar-Morgenstern-Platz 1, Vienna, 1090, Vienna, Austria.

*Corresponding author(s). E-mail(s): freiberger@iiasa.ac.at; grass@iiasa.ac.at; kuhn@iiasa.ac.at; andrea.seidl@univie.ac.at; Wrzaczek@iiasa.ac.at;

This document acts as the Online-Appendix to "Chasing up and locking down the virus: Optimal pandemic interventions within a network" by Freiberger et al. (2022). Besides providing additional and more in-depth results related to the theoretical derivations and numerical example presented in the paper, we discuss the alternative interpretations of our framework. While we investigated a numerical set-up for the spread of an infectious disease and its optimal management across a network of different regions in the main paper, we want to show-case the usage of our framework for the control of a pandemic across a network of different social groups within the same region. Of course in further extensions a combination of both interpretations is possible and the nodes can be used to describe a network of social groups within several different regions, which all potentially interact with each other.

1 Optimality conditions

The complete problem described in equations (14a)-(14i) in Section 3.3 in the main paper is a finite time optimal control model, for which the standard form of the Maximum Principle can be applied (see e.g., Grass et al. [2]). First we formulate the Hamiltonian, which is

$$
\begin{aligned}
\mathcal{H}(\cdot) = &- \sum_{j\in\Omega} \Big[C_M(H_j,t,j) + \mu_H(\overline{H_j},j)H_j \cdot \Psi + C_V(v_j,t,j)\Big] - \\
&- \sum_{(k,j)\in\Omega^2} C_U(u_{k,j},t,k,j) + \sum_{j\in\Omega}\left[\lambda_{S_j}\left(-S_j \cdot \sum_{k\in\Omega}\left[u_{k,j}\cdot \frac{L_k}{S_k+L_k+R_k^L+R_k^D}\right]\right)\right] + \\
&+ \sum_{j\in\Omega}\left[\lambda_{L_j}\left(S_j\cdot\sum_{k\in\Omega}\left[u_{k,j}\cdot\frac{L_k}{S_k+L_k+R_k^L+R_k^D}\right] - \theta_{LH}(j)L_j - \alpha_L(j)L_j - \right.\right.\\
&\left.\left. - \frac{v_j}{L_j+\kappa(S_j+R_j^L)}L_j\right)\right] + \\
&+ \sum_{j\in\Omega}\left[\lambda_{D_j}\left(\frac{v_j}{L_j+\kappa(S_j+R_j^L)}L_j - \alpha_D(j)D_j - \theta_{DH}(j)D_j\right)\right] + \\
&+ \sum_{j\in\Omega}\left[\lambda_{H_j}\Big(\theta_{LH}(j)L_j + \theta_{DH}(j)D_j - \alpha_H(\overline{H_j},j)H_j - \mu_H(\overline{H_j},j)H_j\Big)\right] + \\
&+ \sum_{j\in\Omega}\left[\lambda_{R_j^L}\cdot\alpha_L(j)L_j + \lambda_{R_j^D}\cdot\Big(\alpha_D(j)D_j + \alpha_H(\overline{H_j},j)H_j\Big) + \lambda_{M_j}\cdot\mu_H(\overline{H_j},j)H_j\right] + \\
&+ \sum_{j\in\Omega}\lambda_{\overline{H_j}}\left(\sum_{k\in\Omega}H_k f_H(j,k)\right) + \Lambda\left(\sum_{j\in\Omega}v_j - \overline{V}\right) + \\
&+ \sum_{(k,j)\in\Omega^2}\Big[\overline{\xi_{k,j}}(u_{k,j}-\beta_{k,j}) + \underline{\xi_{k,j}}(\underline{u_{k,j}} - u_{k,j})\Big] + \sum_{j\in\Omega}\Big[\overline{\nu_j}(v_j - \overline{v_j}) + \underline{\nu_j}(\underline{v_j} - v_j)\Big].
\end{aligned}
\tag{1}
$$

λ_{x_j} denotes the adjoint variable of state variable $x_j \in \{S_j, L_j, D_j, H_j, R_j^L, R_j^D, M_j\}$ $(j \in \Omega)$ and Λ denotes the Lagrangian multiplier of the control constraint $\sum_{j\in\Omega} v_j(t) \leq \overline{V}(t)$. $\overline{\xi_{k,j}}, \underline{\xi_{k,j}}, \overline{\nu_j}, \underline{\nu_j}$ $(j,k \in \Omega)$ are the Lagrange multipliers for the upper and lower boundaries of the controls. The lower bound of the transmission rates is denoted by $\underline{u_{k,j}}$.

The first order conditions for the controls read $(j, k \in \Omega)$

$$\frac{\partial \mathcal{H}(\cdot)}{\partial u_{k,j}} = -C_U'(k,j) + \frac{\left(\lambda_{L_j} - \lambda_{S_j}\right) S_j L_k}{S_k + L_k + R_k^L + R_k^D} + \overline{\xi_{k,j}} - \underline{\xi_{k,j}} = 0 \tag{2a}$$

$$\frac{\partial \mathcal{H}(\cdot)}{\partial v_j} = -C_V'(j) - \left(\lambda_{L_j} - \lambda_{D_j}\right) \frac{L_j}{L_j + \kappa(S_j + R_j^L)} + \Lambda + \overline{\nu_j} - \underline{\nu_j} = 0, \tag{2b}$$

with complementary slackness conditions

$$\Lambda \left(\sum_{j \in \Omega} v_j - \overline{V} \right) = 0 \tag{3a}$$

$$\overline{\xi_{k,j}} \left(u_{k,j} - \overline{u_{k,j}}\right) = 0 \quad , \quad (k,j) \in \Omega^2 \tag{3b}$$

$$\underline{\xi_{k,j}} \left(\overline{u_{k,j}} - u_{k,j}\right) = 0 \quad , \quad (k,j) \in \Omega^2 \tag{3c}$$

$$\overline{\nu_j} \left(v_j - \overline{v_j}\right) = 0 \quad , \quad j \in \Omega \tag{3d}$$

$$\underline{\nu_j} \left(\underline{v_j} - v_j\right) = 0 \quad , \quad j \in \Omega. \tag{3e}$$

From the Hamiltonian the adjoint equations can straightforwardly be obtained $(j \in \Omega)$

$$\begin{aligned} \dot{\lambda}_{S_j} &= \rho \lambda_{S_j} + \left(\lambda_{S_j} - \lambda_{L_j}\right) \sum_{k \in \Omega} u_{k,j} \frac{L_k}{S_k + L_k + R_k^L + R_k^D} - \\ &\quad - \sum_{k \in \Omega} \frac{\left(\lambda_{S_k} - \lambda_{L_k}\right) S_k u_{j,k} L_j}{(S_j + L_j + R_j^L + R_j^D)^2} - \frac{\kappa(\lambda_{L_j} - \lambda_{D_j}) L_j v_j}{(L_j + \kappa(S_j + R_j^L))^2} \end{aligned} \tag{4a}$$

$$\begin{aligned} \dot{\lambda}_{L_j} &= \rho \lambda_{L_j} + \sum_{k \in \Omega} \left(\lambda_{S_k} - \lambda_{L_k}\right) S_k u_{j,k} \frac{S_j + R_j^L + R_j^D}{(S_j + L_j + R_j^L + R_j^D)^2} + \\ &\quad + \left(\lambda_{L_j} - \lambda_{D_j}\right) \frac{\kappa v_j (S_j + R_j^L)}{(L_j + \kappa(S_j + R_j^L))^2} - \left(\lambda_{H_j} - \lambda_{L_j}\right) \theta_{LH}(j) - \\ &\quad - \left(\lambda_{R_j^L} - \lambda_{L_j}\right) \alpha_L(j) \end{aligned} \tag{4b}$$

$$\dot{\lambda}_{D_j} = \rho \lambda_{D_j} + \left(\lambda_{D_j} - \lambda_{H_j}\right) \theta_{DH}(j) + \left(\lambda_{D_j} - \lambda_{R_j^D}\right) \alpha_D(j) \tag{4c}$$

$$\begin{aligned} \dot{\lambda}_{H_j} &= \rho \lambda_{h_j} + C_M'(j) + \mu_H \cdot \Psi - \left(\lambda_{R_j^D} - \lambda_{H_j}\right) \alpha_H - \left(\lambda_{M_j} - \lambda_{H_j}\right) \mu_H - \\ &\quad - \sum_{k \in \Omega} \lambda_{\overline{H_k}} f_H(k,j) \end{aligned} \tag{4d}$$

$$\begin{aligned} \dot{\lambda}_{R_j^L} &= \rho \lambda_{R_j^L} - \sum_{k \in \Omega} \left(\lambda_{S_k} - \lambda_{L_j}\right) S_k u_{j,k} \frac{L_j}{(S_j + L_j + R_j^L + R_j^D)^2} + \\ &\quad + \left(\lambda_{L_j} - \lambda_{D_j}\right) \frac{\kappa v_j L_j}{(L_j + \kappa(S_j + R_j^L))^2} \end{aligned} \tag{4e}$$

$$\dot{\lambda}_{R_j^D} = \rho\lambda_{R_j^D} - \sum_{k\in\Omega} \left(\lambda_{S_k} - \lambda_{L_j}\right) S_k u_{j,k} \frac{L_j}{(S_j + L_j + R_j^L + R_j^D)^2} \tag{4f}$$

$$\dot{\lambda}_{M_j} = \rho\lambda_{M_j} \tag{4g}$$

and

$$\lambda_{\overline{H_j}} = -\frac{\partial\mu_H}{\partial\overline{H_j}} H_j(t)\cdot\Psi + \left(\lambda_{R_j^D} - \lambda_{H_j}\right)\frac{\partial\alpha_H}{\partial\overline{H_j}} H_j - \left(\lambda_{H_j} - \lambda_{M_j}\right)\frac{\partial\mu_H}{\partial\overline{H_j}} H_j \tag{5}$$

with the following transversality conditions

$$\lambda_{S_j}(T) = 0 \tag{6a}$$

$$\lambda_{L_j}(T) = \frac{\partial\mathcal{S}(\mathcal{X}(T),T)}{\partial L_j(T)} \tag{6b}$$

$$\lambda_{D_j}(T) = \frac{\partial\mathcal{S}(\mathcal{X}(T),T)}{\partial D_j(T)} \tag{6c}$$

$$\lambda_{H_j}(T) = \frac{\partial\mathcal{S}(\mathcal{X}(T),T)}{\partial H_j(T)} \tag{6d}$$

$$\lambda_{R_j^L}(T) = 0 \tag{6e}$$

$$\lambda_{R_j^D}(T) = 0 \tag{6f}$$

$$\lambda_{M_j}(T) = 0. \tag{6g}$$

2 Interpretation of shadow prices

Solving for the shadow prices (i.e., the adjoint variables) allows us to identify the interconnected channels that determine the "values" (from an epidemiological perspective) of individuals within the different compartments. The shadow price of a susceptible at node j reads

$$\lambda_{S_j}(t) = \int_t^T e^{-\rho(s-t)} \Bigg(\underbrace{\left(\lambda_{L_j} - \lambda_{S_j}\right)\sum_{k\in\Omega} u_{k,j}\frac{L_k}{S_k + L_k + R_k^L + R_k^D}}_{=(i)} - \\ - \underbrace{\frac{L_j}{(S_j + L_j + R_j^L + R_j^D)^2}\sum_{k\in\Omega} u_{j,k}\left(\lambda_{L_k} - \lambda_{S_k}\right) S_k}_{=(ii)} - \\ - \underbrace{\frac{\kappa v_j}{(L_j + \kappa(S_j + R_j^L))^2}(\lambda_{D_j} - \lambda_{L_j}) L_j}_{=(iii)} \Bigg)\, dt. \tag{7}$$

The value of a susceptible, as given by λ_S, aggregates the discounted value of three effects. (i) captures the expected loss of an additional (marginal) susceptible at node j becoming infected and turning into a light case. The expected loss consist of the vale of a susceptible getting infected and turning into a light case, $\lambda_{L_j} - \lambda_{S_j} < 0$,[1] weighted with the (aggregate) probability of getting infected through a contact from any node in the network. The other two effects capture second-order effects. Specifically, (ii) describes the expected value of a marginal susceptible in lowering the share of light cases in the population at node j and, thus, the aggregate risk of a light case at j infected a susceptible at any other node k. (iii) measures that an additional susceptible at node j makes it harder to identify a light case via testing. The change in probability of finding a light case in the relevant population via testing is then weighted by the value of a light case being detected, $\lambda_{D_j} - \lambda_{L_j} > 0$.[2]

The shadow price of light cases at node j is structured similarly and reads

$$\begin{aligned}\lambda_{L_j}(t) = \int_t^T e^{-\rho(s-t)} \Bigg[&\left(\lambda_{H_j} - \lambda_{L_j}\right) \theta_{LH}(j) + \left(\lambda_{R_j^L} - \lambda_{L_j}\right) \alpha_L(j) + \\ &+ \frac{S_j + R_j^L + R_j^D}{(S_j + L_j + R_j^L + R_j^D)^2} \sum_{k \in \Omega} \left(\lambda_{L_k} - \lambda_{S_k}\right) S_k u_{j,k} + \\ &+ \frac{\kappa v_j (S_j + R_j^L)}{(L_j + \kappa(S_j + R_j^L))^2} \left(\lambda_{D_j} - \lambda_{L_j}\right) \Bigg] dt + e^{-\rho(T-t)} \frac{\partial \mathcal{S}(\mathcal{X}(T), T)}{\partial L_j(T)}.\end{aligned} \tag{8}$$

The first two terms in the integral are direct effects. They contain the value of a light case escalating to a heavy case (moving to the H_j compartment) and the value of a light case recovering from the infection (moving to the R_j^L compartment). Both terms are multiplied by their respective rate of occurrence. The third term captures the effect of light cases at node j on overall infections within node j and into other nodes. The fourth term captures the effect on the detection of light cases. The term outside the integral is the discounted effect of light cases on the costs that arise after the planning horizon.

Moving on to the shadow price of detected cases at node j, we can derive the following expression:

$$\begin{aligned}\lambda_{D_j}(t) = \int_t^T e^{-\rho(s-t)} \left[\left(\lambda_{H_j} - \lambda_{D_j}\right) \theta_{DH}(j) + \left(\lambda_{R_j^D} - \lambda_{D_j}\right) \alpha_D(j) \right] dt + \\ + e^{-\rho(T-t)} \frac{\partial \mathcal{S}(\mathcal{X}(T), T)}{\partial D_j(T)}.\end{aligned} \tag{9}$$

[1] The negative sign is readily verified for an interior optimum from the FOC (15a) in the main paper.

[2] The positive sign is readily verified for an interior optimum from the FOC (15b) in the main paper.

The two terms within the integral capture once again the values of a detected individual developing heavy symptoms or recovering from the disease (in this case moving to the R_j^D compartment, however). Again, the values of the two transitions are multiplied by their respective arrival rates. Note that for detected cases these may differ from the respective rates in case of light cases. Specifically, the scope for monitoring and early on treatment in case of detection will lead to a higher (lower) rate of recovery (disease escalation), implying in turn a higher value of a detected case. As detected cases are quarantined, there are no knock-on effects on the process of infection, nor on the success of testing. The term outside the integral again corresponds to the discounted effect on costs after the planning horizon.

The shadow price of heavy cases at node j reads

$$\lambda_{H_j}(t) = \int_t^T e^{-\rho(s-t)} \Bigg[\underbrace{-C_M'(j) - \mu_H \cdot \Psi}_{=(i)} + \underbrace{\left(\lambda_{R_j^D} - \lambda_{H_j}\right) \alpha_H + \left(\lambda_{M_j} - \lambda_{H_j}\right) \mu_H}_{=(ii)} + \\ + \underbrace{\sum_{k\in\Omega} \lambda_{\overline{H_k}} f_H(k,j)}_{=(iii)} \Bigg] dt + e^{-\rho(T-t)} \frac{\partial \mathcal{S}(\mathcal{X}(T),T)}{\partial H_j(T)}. \tag{10}$$

The integral aggregates the discounted value of three effects: (i) counts the costs of an additional heavy case at node j, consisting of medical treatment costs and the value of a lost life (weighted by the death rate). (ii) values the the transitions to recovery (in this case into the $\lambda_{R_j^D}$ compartment) and death. Note that while (i) values mortality by the value of a lost life, (ii) focuses on the value change due to a heavy case moving to the M_j compartment. Note that these two effects typically differ in their sign. (iii) assigns a value to heavy cases at node j based on their utilisation of hospital that is no longer available at other nodes. The term outside the integral captures the discounted effect on the costs after the planning horizon.

The shadow prices of recovered cases (light or diagnosed) at node j

$$\lambda_{R_j^L}(t) = \int_t^T e^{-\rho(s-t)} \Bigg(- \frac{L_j}{(S_j + L_j + R_j^L + R_j^D)^2} \sum_{k\in\Omega} \left(\lambda_{L_k} - \lambda_{S_k}\right) S_k u_{j,k} + \\ + \left(\lambda_{D_j} - \lambda_{L_j}\right) \frac{\kappa v_j L_j}{(L_j + \kappa(S_j + R_j^L))^2} \Bigg) dt, \tag{11}$$

$$\lambda_{R_j^D}(t) = \int_t^T e^{-\rho(s-t)} \left(- \frac{L_j}{(S_j + L_j + R_j^L + R_j^D)^2} \sum_{k\in\Omega} \left(\lambda_{L_k} - \lambda_{S_k}\right) S_k u_{j,k} \right) ds \tag{12}$$

consist of indirect effects, similar to the ones for susceptibles. Recovered cases at node j lower the probability of infection from j to any other node. Light

recovered cases also tend to lower the probability of detection at this node, an effect that is absent for recovered detected cases, implying they tend to be of a higher value.

Finally, the shadow price of the aggregated variable $\overline{H_j}$ can be derived as

$$\lambda_{\overline{H_j}}(t) = -\frac{\partial \mu_H}{\partial \overline{H_j}} H_j(t) \cdot \Psi + \left(\lambda_{R_j^D} - \lambda_{H_j}\right) \frac{\partial \alpha_H}{\partial \overline{H_j}} H_j + \left(\lambda_{M_j} - \lambda_{H_j}\right) \frac{\partial \mu_H}{\partial \overline{H_j}} H_j. \tag{13}$$

The first part counts the value of additional lives lost due to the increase in mortality within congested hospitals; the second term values the decline in the recovery rate for heavy cases within congested hospitals; and the third term values the increase in the mortality rate from perspective of the epidemiological system (and disregarding the value of lives lost.

3 Additional results for the regional framework

3.1 Summary tables for numerical results

	S	R_L	R_D	M	Agg. Costs	t=100	t=200	t=300	t=400
Region 1	0.03429	0.28638	0.0029	0.00977	**Total**	70.23	71.91	71.92	71.92
	(10.29%)	(85.91%)	(0.87%)	(2.93%)	VOL lost	69.64	71.28	71.29	71.29
					Medical	0.59	0.63	0.63	0.63
Region 2	0.03796	0.28286	0.0027	0.00981	**Total**	70.31	72.23	72.24	72.24
	(11.39%)	(84.86%)	(0.81%)	(2.94%)	VOL lost	69.74	71.61	71.62	71.62
					Medical	0.57	0.62	0.62	0.62
Region 3	0.03837	0.28247	0.00268	0.00982	**Total**	70.32	72.27	72.28	72.28
	(11.51%)	(84.74%)	(0.8%)	(2.95%)	VOL lost	69.74	71.64	71.65	71.65
					Medical	0.57	0.62	0.62	0.62
Total	0.11062	0.85171	0.00828	0.0294	**Total**	210.86	216.41	216.44	216.44
					VOL lost	209.13	214.53	214.56	214.56
					Medical	1.73	1.88	1.88	1.88

Table 1 Terminal states at $T = 400$ of the population (susceptibles S, recovered light R_L cases, diagnosed/heavy cases R_D, and deceased M) and cumulative costs in units of GDP per capita per day at days 100, 200, 300 and 400 in the "Uncontrolled" case.

	S	R_L	R_D	M	Agg. Costs	t=100	t=200	t=300	t=400
Region 1	0.09195	0.23097	0.00865	0.00154	**Total**	19.92	27.22	30.26	31.52
	(27.58%)	(69.29%)	(2.6%)	(0.46%)	VOL lost	5.07	7.94	10.12	11.27
					Medical	0.23	0.36	0.46	0.51
					Lockdown	14.63	18.92	19.69	19.74
Region 2	0.13344	0.19112	0.00715	0.00127	**Total**	7.18	15.74	20.6	22.58
	(40.03%)	(57.34%)	(2.14%)	(0.38%)	VOL lost	1.62	4.5	7.51	9.28
					Medical	0.07	0.2	0.34	0.42
					Lockdown	5.49	11.04	12.75	12.88
Region 3	0.13833	0.18643	0.00697	0.00124	**Total**	5.34	13.69	18.76	20.83
	(41.5%)	(55.93%)	(2.09%)	(0.37%)	VOL lost	1.32	4.11	7.2	9.05
					Medical	0.06	0.19	0.33	0.41
					Lockdown	3.96	9.4	11.23	11.37
Total	0.36372	0.60852	0.02277	0.00405	**Total**	32.45	56.65	69.63	74.93
					VOL lost	8.01	16.55	24.83	29.6
					Medical	0.36	0.75	1.12	1.34
					Lockdown	24.07	39.36	43.67	44.0

Table 2 Terminal states of the population (susceptibles S, recovered light R_L cases, diagnosed/heavy cases R_D, and deceased M) and cumulative costs in units of GDP per capita per day at days 100, 200, 300 and 400 in the "No testing" case.

	S	R_L	R_D	M	Agg. Costs	t=100	t=200	t=300	t=400
Region 1	0.10436	0.21329	0.01407	0.00147	**Total**	19.25	25.39	28.17	29.12
	(31.31%)	(63.99%)	(4.22%)	(0.44%)	VOL lost	5.02	7.76	9.83	10.7
					Medical	0.23	0.35	0.44	0.48
					Lockdown	14.0	17.28	17.89	17.93
					Testing	0.0	0.01	0.01	0.01
Region 2	0.14713	0.16305	0.02176	0.00119	**Total**	7.06	14.92	18.93	20.3
	(44.14%)	(48.92%)	(6.53%)	(0.36%)	VOL lost	1.63	4.55	7.41	8.66
					Medical	0.07	0.21	0.34	0.39
					Lockdown	5.36	10.15	11.17	11.22
					Testing	0.0	0.01	0.02	0.03
Region 3	0.15189	0.15788	0.02219	0.00116	**Total**	5.35	13.17	17.32	18.74
	(45.57%)	(47.36%)	(6.66%)	(0.35%)	VOL lost	1.34	4.21	7.14	8.43
					Medical	0.06	0.19	0.32	0.38
					Lockdown	3.95	8.77	9.83	9.89
					Testing	0.0	0.01	0.02	0.03
Total	0.40338	0.53422	0.05802	0.00382	**Total**	31.66	53.49	64.42	68.16
					VOL lost	7.98	16.52	24.38	27.79
					Medical	0.36	0.74	1.1	1.26
					Lockdown	23.31	36.21	38.89	39.04
					Testing	0.01	0.02	0.05	0.07

Table 3 Terminal states of the population (susceptibles S, recovered light R_L cases, diagnosed/heavy cases R_D, and deceased M) and cumulative costs in units of GDP per capita per day at days 100, 200, 300 and 400 in the "Ineffective testing" case.

	S	R_L	R_D	M	Agg. Costs	t=100	t=200	t=300	t=400
Region 1	0.18006	0.11874	0.03356	0.00098	**Total**	15.07	18.25	18.8	18.82
	(54.02%)	(35.62%)	(10.07%)	(0.29%)	VOL lost	4.49	6.62	7.12	7.14
					Medical	0.2	0.3	0.32	0.32
					Lockdown	10.37	11.33	11.34	11.34
					Testing	0.0	0.01	0.01	0.02
Region 2	0.22686	0.07266	0.03313	0.00068	**Total**	6.18	10.12	10.81	10.84
	(68.06%)	(21.8%)	(9.94%)	(0.2%)	VOL lost	1.78	4.29	4.93	4.95
					Medical	0.08	0.2	0.22	0.23
					Lockdown	4.32	5.62	5.64	5.64
					Testing	0.0	0.01	0.02	0.02
Region 3	0.23081	0.06992	0.03195	0.00065	**Total**	5.31	9.32	10.02	10.05
	(69.24%)	(20.98%)	(9.59%)	(0.2%)	VOL lost	1.55	4.1	4.74	4.77
					Medical	0.07	0.19	0.22	0.22
					Lockdown	3.68	5.03	5.04	5.04
					Testing	0.0	0.01	0.02	0.02
Total	0.63773	0.26132	0.09864	0.00231	**Total**	26.56	37.69	39.63	39.7
					VOL lost	7.83	15.01	16.79	16.85
					Medical	0.36	0.68	0.76	0.77
					Lockdown	18.38	21.98	22.03	22.03
					Testing	0.01	0.02	0.05	0.06

Table 4 Terminal states of the population (susceptibles S, recovered light R_L cases, diagnosed/heavy cases R_D, and deceased M) and cumulative costs in units of GDP per capita per day at days 100, 200, 300 and 400 in the "Effective testing" case.

4 Network of social groups

4.1 Parametrisation

For this presentation we divide the population of a single region into three groups:

Blue-collar workers represent employees in the retail sector or medical workers. They are no more vulnerable than the general population, but their work requires higher interaction with other people. Therefore reducing the transmission rates for them is more costly as they cannot work from home. Stay-at-home orders for these worker therefore imply high economic costs.
White-collar workers represent the population with office jobs. For them the costs for reducing the transmission rate is lower, as they can switch to home office and thereby reduce contacts without exceedingly high costs.
Vulnerable population represents older people and people with predispositions. This group faces a higher probability of developing a severe course of the disease and in that case also face a higher mortality rate and lower recovery rate.

The groups are assumed to be of equal size, a stylization that allows us to identify better the role of the outlined heterogeneity for policy-making. We continue to set the regeneration rates α_L and α_D equal to 1/15. For the calibration of the mortality and escalation rates we use age-specific data from Brazeau et al. (2020) [1] and Souris and Gonzalez (2020)[3]. While blue and white collar workers face a 5.6% probability of developing a severe course, this value increases to 15.7% for the vulnerable group. Similarly, the vulnerable face mortality risk in the presence of full ICU capacity of 18.5%, whereas this risk is 3.0% for workers. Given these probabilities we can again derive the corresponding rates given the average dwell time in the light-case (15 days) and heavy-case (12 days) states as shown in (14) - (15):

$$\theta_{LH} = \theta_{DH} = \begin{pmatrix} 1 - 0.944^{\alpha_L} \\ 1 - 0.944^{\alpha_L} \\ 1 - 0.843^{\alpha_L} \end{pmatrix} = \begin{pmatrix} 0.003859 \\ 0.003859 \\ 0.011290 \end{pmatrix} \tag{14}$$

$$\overline{\mu_H} = \begin{pmatrix} 0.02989/12 \\ 0.02989/12 \\ 0.18550/12 \end{pmatrix} = \begin{pmatrix} 0.002491 \\ 0.002491 \\ 0.015458 \end{pmatrix}. \tag{15}$$

While the uncontrolled transmission rates are assumed to be uniform between and within all groups, the economic costs differ as described above.

We propose the following cost matrix:

$$\overline{C_u} = \begin{pmatrix} 0.35 & 0.2 & 0.2 \\ 0.2 & 0.15 & 0.1 \\ 0.2 & 0.1 & 0.1 \end{pmatrix} \tag{16}$$

This matrix should reflect, that it is most difficult and therefore most costly to avoid transmissions between blue-collar workers (0.35), followed by white collar workers (0.15), who can be more easily required to work from home. We also attribute costs to the transmission reductions of the most vulnerable population group (consisting mostly of the older part of the total population already in retirement), as (i) it still consists partly of individuals still in working ages (with other characteristic like pre-existing health conditions making them vulnerable), and (ii) to reflect some social costs of putting individuals increasingly into isolation. For the costs between the different groups, we assumed that contact with blue-collar workers is harder to eliminate for both other groups, since people still shop for groceries, etc. and some level of interaction is hardly avoidable. On the other hand, we assume that contacts between white-collar workers and the vulnerable population can be reduced relatively cheap.

Altogether, we make the following assumptions:

(S1) The social groups are identical in size and the sum of them is normalized to 1, i.e. $N_i(t) = 1/3$ for all t and $i \in \Omega$.
(S2) Epidemiological and cost parameters differ across groups according to (14)-(16).
(S3) Hospital capacities are shared between the social groups, i.e. $f_H(j,k) \equiv 1$.
(S4) Testing capacities are shared between the social groups.

Finally, for the purpose of this analysis, we focus on a single scenario in respect to testing effectiveness. Assuming a relatively effective process of contact tracing, allowing for targeted testing, we set $\kappa = 0.1$. The initial share of infected individuals is assumed to be 0.1% for all groups. All the other parameters are chosen as they were in the main paper Freiberger et al. (2022) and are summarised in (14) - (15) and Table 5.

Economic Parameters		Epidemiological Parameters	
n	3	$\mathcal{I}_0$	2.5
ρ	0.0	α_L	1/15
$\overline{N}$	$\sum_i N_i(0) = 1.0$	α_D	1/15
GDP	$50000[\$/c]/365[d] = 137.0[\$/c/d]$	θ_{LH}	$1-(1-0.0065/0.15)^{\alpha_L} = 0.002948$
Ψ	$10^6[\$]/GDP = 7300[GDP]$	θ_{DH}	$1-(1-0.0065/0.15)^{\alpha_D} = 0.002948$
ICU - Cap	$\overline{N} \cdot 0.0003$	$\overline{\alpha_{H,j}}$	$1/12 - \overline{\mu_{H,j}} = 0.07083$
$\overline{C^i_M}$	$(2/3 \cdot 190\$ + 1/3 \cdot 1330\$)/GDP = 4.161[GDP]$		
$\overline{C^i_V}$	$5\$/GDP = 0.0365[GDP]$		

Table 5 Parameters for the numerical discussion of scenarios with heterogeneous social groups.

In the following we present the analysis for three scenarios: (i) "Uncontrolled", (ii) "No Testing" and (iii) "Testing (at $\kappa = 0.1$)".

4.2 Uncontrolled development

Figure 1 presents the most important epidemiological states for an uncontrolled pandemic: susceptibles, light cases and heavy cases. The blue solid line denotes the corresponding state for blue-collar workers, the red one that for white-collar workers and the yellow one that for the vulnerable population. The dashed black line denotes the total number of light and heavy cases, respectively. In the absence of any controls and due to the homogeneous transmission rates between all groups the development of susceptibles and light cases is nearly identical. However the number of heavy cases in the vulnerable population is significantly higher due to their increased escalation rate. Overall, the pandemic is short but hefty, as follows from the fact that ICU capacity, denoted by the dotted green line in the panel is exceeded by the total number of heavy cases (dashed black line) over almost the full course of the pandemic. This causes a high number of deaths, which, as we will see, could have been prevented by measures aimed at controlling the disease.

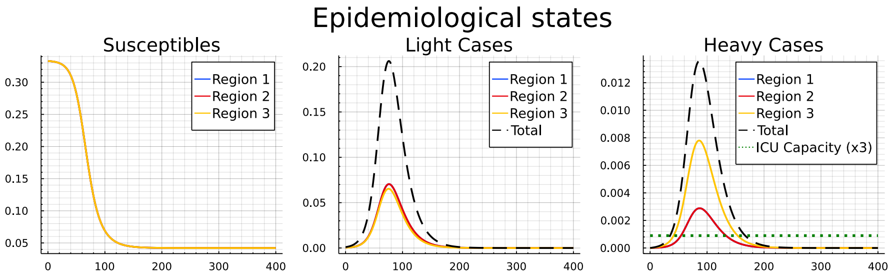

Fig. 1 Pandemic development in the "Uncontrolled" case (with social groups).

Table 6 includes a summary of the terminal outcomes of the pandemic after $T = 400$ for each social group as well as for the total population (as percentages of each group resp. the total initial population size). The first four columns show the percentage of people, who have not been infected (S), recovered from the disease (R_L and R_D), and died (stage M), respectively. The final four columns show (from the left) the cumulated costs of the pandemic up until day 100, 200, 300 and 400 (i.e. end time), respectively. As we know from Figure 1 the pandemic is raging mainly over the first 100 days, and has "burnt out" by day 200. Thus, costs do not change afterwards. The Table also mirrors that the development within blue and white collar workers is identical (due to their identical epidemiological parameters). In the vulnerable population, however, mortality is nearly threefold and similarly costs (mostly arising from value of lives lost) is increased by around 170% compared to the other two social groups.

Turning to infections, we see that in each region only around 12.6% of the population remain susceptible and more than 81% are either recovered light or heavy cases. More than 6% of the population have died during the pandemic, predominantly as a result of an overwhelmed health sector (recall the right panel in figure 1).

**Uncontr.**	Epidemiological states at $t = 400$				Costs until t (in GDP p.c.p.d.)			
**Case**	S	R_L	R_D	M	t=100	t=200	t=300	t=400
Blue-collar	12.64%	82.58%	0.77%	4.0%	61.7	98.23	98.25	98.25
White-collar	12.64%	82.58%	0.77%	4.0%	61.7	98.23	98.25	98.25
Vulnerable	12.64%	74.71%	1.68%	10.97%	171.75	268.93	269.15	269.15
Total	12.64%	79.96%	1.08%	6.33%	295.16	465.38	465.65	465.65
**No**	Epidemiological states at $t = 400$				Costs until t (in GDP p.c.p.d.)			
**Testing**	S	R_L	R_D	M	t=100	t=200	t=300	t=400
Blue-collar	49.91%	47.26%	2.64%	0.08%	13.15	33.92	44.6	47.11
White-collar	53.98%	43.41%	2.42%	0.08%	9.27	21.73	33.25	38.12
Vulnerable	80.97%	16.22%	2.21%	0.51%	5.39	10.41	16.85	22.91
Total	61.62%	35.63%	2.42%	0.22%	27.81	66.06	94.7	108.14
**Ineffect.**	Epidemiological states at $t = 400$				Costs until t (in GDP p.c.p.d.)			
**Testing**	S	R_L	R_D	M	t=100	t=200	t=300	t=400
Blue-collar	75.46%	12.82%	11.68%	0.04%	4.35	6.86	7.26	7.29
White-collar	76.73%	15.7%	7.53%	0.04%	7.97	13.56	14.09	14.13
Vulnerable	84.44%	10.7%	4.45%	0.42%	6.06	13.48	16.9	17.31
Total	78.87%	13.07%	7.89%	0.17%	18.37	33.9	38.26	38.74

Table 6 Summary and comparison of the endstates of the pandemic and the costs of the pandemic across the three different social groups for the three scenarios analysed. Endstates are given in percentage of the initial population size of each region, resp. the total population. Costs are given in units of GDP per capita per day (GDP p.c.p.d.).

4.3 Controlled development

In this section we compare the development across two scenarios in which the social planner is assumed to have increasing capabilities of controlling the disease.

4.3.1 Epidemic progression

Figure 2 allows for comparison of the epidemiological development of the pandemic over time between the no-testing and testing case.[3] The first column illustrates the development for each social group (colored lines) and in total (black dashed line) when tests are not available. The following conclusions are evident. Compared to the uncontrolled development, first, the duration of the pandemic is much longer and basically stretches until a vaccine becomes universally available at the end of the time horizon. Second, the main focus of the measures lies on protecting the vulnerable population, which remains susceptible for the most part until a vaccine becomes available. Blue-collar workers on the other hand show the highest number of infections. The total number of infections increases within the first 60 day and than plateaus for another 150 day. Consequently the number decreases slowly over time before a slight resurgence occurs towards the end of the time horizon in anticipation of the vaccine becoming available.

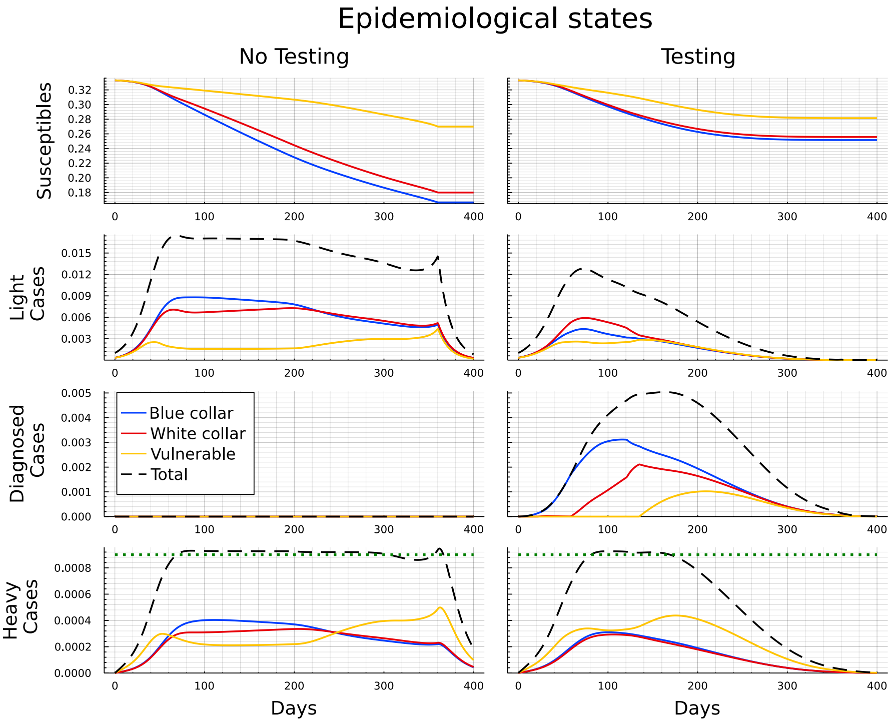

Fig. 2 Comparison of the pandemic development for the "No testing" and "Testing" case.

[3] To keep the scales for each state variable the same for all scenarios, we decided to present the uncontrolled development of the pandemic separately, since the peak number of light and heavy cases are tenfold the number when any controls are available.

The total number of heavy cases follows the light case dynamics within a small delay and ICU capacities are under full usage from day 70 right until the vaccine becomes available. The composition of heavy cases however varies over time. Within the first surge of the pandemic the largest share of heavy cases consists of vulnerable individuals, after 60 days, however, blue-collar workers constitute the largest group. The higher number of infections within this group leads to higher hospitalisation numbers, despite the significantly smaller escalation rates compared to the vulnerable population. The profile of white-collar workers is relatively similar to the one of blue-collar workers, but on a slightly lower level for some time in the middle of the pandemic. After 240 days the vulnerable population again constitutes the majority of hospitalised individuals until a vaccine becomes available.

The second part in Table 6 shows the endstates of all relevant variables and the arising costs until different points in time for optimal target transmission rates but without testing. While for the most vulnerable the share of deaths is reduced from nearly 11% to 0.51%, mortality also drops for workers from 4% to 0.08%. This is possible despite about 50% in each of the working population groups getting infected at some point during the pandemic. Despite significant economic costs now arising due to reduction in the transmission rates, the total costs of the pandemic are much reduced and more evenly spread across its duration. While the total costs within the workers groups drop by some 53% and 62% respectively, the most significant improvement occurs within the most vulnerable population. The relatively low costs of transmission rate reductions turn out to also be highly cost-effective with respect to value of lives lost. In total the costs within this group are below 10% of the one resulting from an uncontrolled development.

After all (as for the regional groups in the main paper), all these features correspond to "classical" curve-flattening as an optimal policy to contain the number of deaths by avoiding excessive ICU congestion while at the same time containing the costs of shutting down the economy.

Now considering the right columns in Figure 2, we can identify a significant impact of effective testing becoming available. While the number of susceptibles over time remains roughly the same for the most vulnerable group, significantly more individuals of both worker groups remain susceptible over time. Table 6 shows the number of susceptibles at the time a vaccination becomes available increases from around 50% to some 75% for both blue and white-collar workers. This dynamics originate in the profile of undetected light cases, which drives the infections of susceptibles. Although the total number of light cases peak around the same time as without tests, the peak is on a significantly lower level and the number of undetected light cases continuously decreases afterwards until they reach similar levels as in the beginning around day 300. The development within the individual groups is rather homogeneous with an intermediate period of slightly raised undetected cases within the white collar workers.

The diagnosed cases reflect the testing strategy (which we will discuss later on) and show a sequential uptake in detections first within the blue-collar workers, followed by the white-collar workers and the vulnerable population.

Lastly the profiles of heavy cases also significantly change, if effective testing is introduced. The total number of hospitalisations still implies full ICU usage after day 80, however, after 180 days the ICU does not have to work under full capacity anymore, since the hospitalisation numbers continuously drop afterwards. Interestingly the number of vulnerable people with heavy progressions of the disease is slightly higher in comparison to the scenario without testing, during a long stretch. On the other hand significantly fewer people from the worker groups need hospital treatment.

4.3.2 Optimal pandemic controls

In this section we want to discuss the optimal target transmission rates and testing strategy (in case tests are available). Figure 3 shows the optimal target transmission rates in case test are available (blue dashed-dotted) or not (red solid). The black dashed lines indicate the uncontrolled transmission rates (upper bound) and the lowest possible transmission rate.

In case of "No testing" (red solid lines) Figure 3 shows that most lockdown measures are introduced only gradually as the numbers of light cases increases with the lowest targeted transmission rates after 70 days, roughly the same time at which the cases in intensive care reach the capacity threshold. Notably, however, transmissions from blue and white-collar workers on the vulnerable are reduced to the lowest possible value already after 45 days and remain in place for most of the time[4]. With this protection in place, the interactions/transmissions within the most vulnerable group remain less restricted. Comparing the transmission targets across the two groups of workers, we see that lockdown measures are stricter for white-collar workers, reflecting their lower cost of restriction. The resulting higher infection rates among blue-collar workers then imply that they are exposed to stronger restrictions vis-a-vis white-collar workers. Notably, however, this does not apply to restrictions vis-a-vis the vulnerable. Here, restrictions remain in place for a longer duration on white-collar workers. This results from the number of light cases having equalized across the two groups of workers towards the end of the pandemic, the lower cost of restrictions towards the vulnerable on the part of white collar workers then becomes the dominant factor for the extended reduction in transmissions.

Due to their very low infection numbers, potential transmissions from the vulnerable group on the workers groups are hardly necessary. Hence the target transmission rates are relatively close to their uncontrolled values for nearly the

[4]Again under the assumption that uni-directional measures are possible, the burden of restrictions falls on the groups of workers as opposed to the group of the vulnerable. This reflects the much larger number of infected among the workers.

entire duration of the pandemic. Furthermore note that the (nearly total) isolation of vulnerable people from the two worker groups allows for less restricted interaction within the vulnerable population.

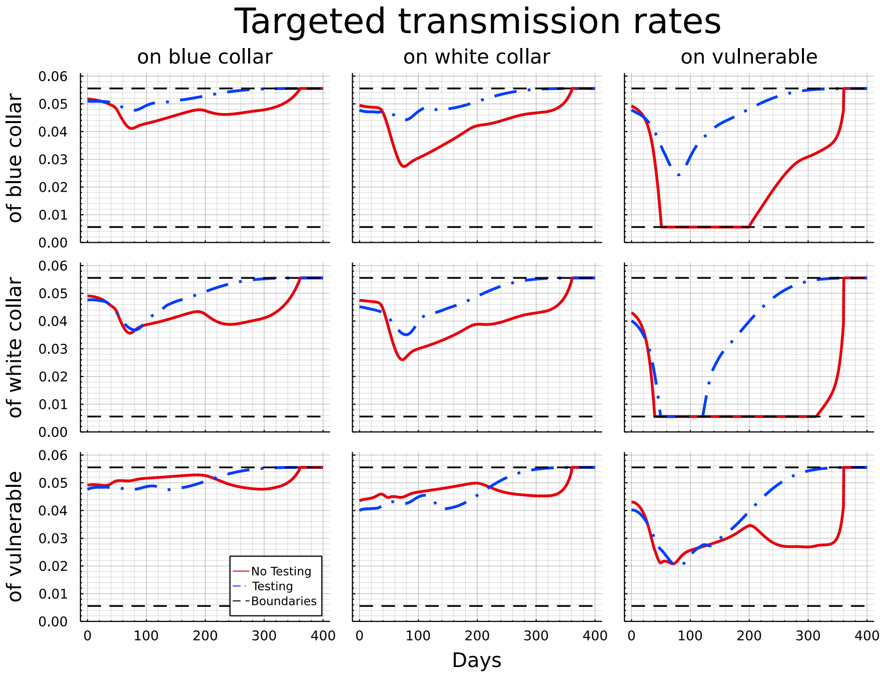

Fig. 3 Optimal target transmission rates for the "No testing" and "Testing" case.

The introduction of efficient testing now allows for significant changes in the optimal lockdown strategies. Interestingly the target transmission rates within the first 20-40 days are on the same level or even more restrictive compared to the case of no available testing. Subsequently almost all restriction can be weakened faster and more pronounced. After around 300 days all transmission almost return to their uncontrolled levels. Especially the total isolation of the vulnerable population from the blue-collar workers is not necessary anymore. This target transmission rate still drops to its lowest value around the time the hospitalisation number reaches the ICU-capacity to avoid further infections and consequently heavy cases. Meanwhile the transmission rate from white-collar workers on the vulnerable population is reduced to the lowest value possible with twofold reasoning: (i) the lower costs of reducing interactions and transmissions from white-collar workers compared to blue-collar workers makes full isolation still cost-effective. (ii) the number of undetected light cases is lower for the blue-collar workers, as testing picks up first within this group as Figure 4 shows.

The strongly limited testing capacity in the beginning is used for tests in the blue-collar group solely. After 50 days an increasing share is allocated to

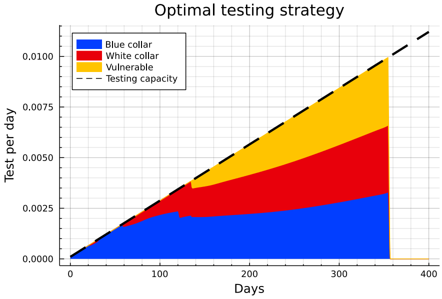

Fig. 4 Optimal allocation of tests between different social groups.

the white-collar workers, with testing within the vulnerable group only picking up after 130 days. Consequently the distribution of tests between the different groups becomes increasingly uniform. We can summarise the optimal strategy of lockdown and testing as follows:

- Protect the vulnerable population from infection by other groups, so interactions within the vulnerable can still be allowed to some degree without risking to many fatalities.
- Allocate limited tests predominantly to the group, where transmission reductions are most costly. With the decreased number of undetected cases, relatively high transmission rates from and within this group can be allowed (the transmission rates for blue-collar workers on the other groups are actually lower than the same rates of white-collar workers, see Figure 3).
- As testing capacities increase, allocate tests to groups according to their respective lockdown costs.

Or to keep it within one sentence: "Isolate the vulnerable, test the economic interconnected groups!".

Finally we discuss the cost composition for the "No testing" and "Testing" case. Figure 5 illustrate the cost flows over time. First of all, note that the costs for testing and medical treatment are rather negligible compared to the costs resulting from lockdowns and value of lives lost. In case of no available testing, the dominating part of costs arise from reducing transmission from blue-collar workers on other groups (which is also the most expensive in our parametrisation), followed by the transmission reduction costs for white-collar workers. This is due to the fact that many blue- and white-collar workers are allowed to get infected (light cases + diagnosed cases) as they exhibit a lower risk to suffer a heavy course and at the same time have high lockdown costs.

The value of lives lost originates especially from deaths within the most vulnerable group and remains on similar levels for the most part of the pandemic. Also in case tests become available the value of lives lost still stays within a similar range, while in aggregation dropping by some 25% (see also table 7).

Meanwhile the total costs are more substantially reduced at all points in time through lowering costs for transmission reductions by the blue and white-collar workers (coinciding with the decreased lockdown efforts in Figure 3).

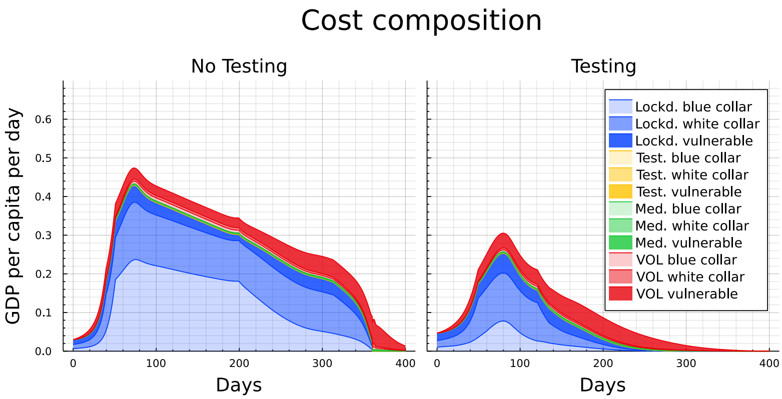

Fig. 5 Origins of costs for the "No testing" case (left) and the "Testing case" (right).

4.3.3 Scenario comparisons

Finally we want to compare all different scenarios by some key values summarised in Table 7.

The uncontrolled solution performs very bad both in terms of total deaths (6.3% of the population) and total costs (arising only from costs corresponding to treatment and lost lives). The other two solutions perform much better. In the case without testing only 0.22% of the population die at less than one fourth of the costs compared to the uncontrolled solution. With optimal testing mortality can be further decreased to 0.17% of the population, while costs can be lowered by an additional two thirds at the same time. The last part of

		Uncontrolled	No Testing	Testing
Endstates (t=400)	S	0.12636	0.61623	0.78874
	R_L	0.79957	0.35631	0.13071
	R_D	0.01075	0.02423	0.07887
	M	0.06328	0.00223	0.00166
Agg. Costs in GDPPCPD (t=400)	Total	465.65	108.14	38.74
	VOL lost	461.95	16.29	12.1
	Lockdown	0.0	90.53	25.76
End of Spread (days)		203.0	360.0	336.0
End ICU (days)		167.0	366.0	169.0
End Lockdown (days)		0.0	360.0	320.0

Table 7 Comparison of the 4 (main) scenarios.

Table 7 shows that a short but ravaging pandemic can be attenuated at the cost of extending its duration until a vaccine becomes universally available. Furthermore the introduction of efficient testing does not only allow to advance the lifting of all lockdown measures by some 40 days, but also reduces the

Springer Nature 2021 LaTeX template

burden in the ICU, which has to act under full capacity usage for roughly 200 fewer days.

References

[1] Brazeau, N., Verity, R., Jenks, S., Fu, H., Whittaker, C., Winskill, P., ... & Okell, L. (2020). Report 34: COVID-19 infection fatality ratio: estimates from seroprevalence.

[2] Grass, D., Caulkins, J.P., Feichtinger, G., Tragler, G., Behrens, D. (2008) Optimal Control of Nonlinear Processes: With Applications in Drugs, Corruptions, and Terror. Springer-Verlag, Berlin.

[3] Souris, M., & Gonzalez, J. P. (2020). COVID-19: Spatial analysis of hospital case-fatality rate in France. PloS one, 15(12), e0243606.
